# Good Vibrations Report on the DNA Quadruplex Binding of an Excited State Amplified Ruthenium Polypyridyl IR Probe

**DOI:** 10.1021/jacs.3c06099

**Published:** 2023-09-22

**Authors:** Mark Stitch, Davide Avagliano, Daniel Graczyk, Ian P. Clark, Leticia González, Michael Towrie, Susan J Quinn

**Affiliations:** † School of Chemistry, University College Dublin, Dublin, D04 V1W8, Ireland; ‡ Institute of Theoretical Chemistry, Faculty of Chemistry, University of Vienna, Währingerstr. 19, 1090 Vienna, Austria; § Department of Chemistry, Chemical Physics Theory Group, University of Toronto, 80 St. George St., Toronto, Ontario M5S 3H6, Canada; ∥ Central Laser Facility, STFC Rutherford Appleton Laboratory, Harwell Science and Innovation Campus, Didcot, Oxfordshire OX11 0QX, U.K.; ⊥ Vienna Research Platform on Accelerating Photoreaction Discovery, University of Vienna, Währingerstr. 19, 1090 Vienna, Austria

## Abstract

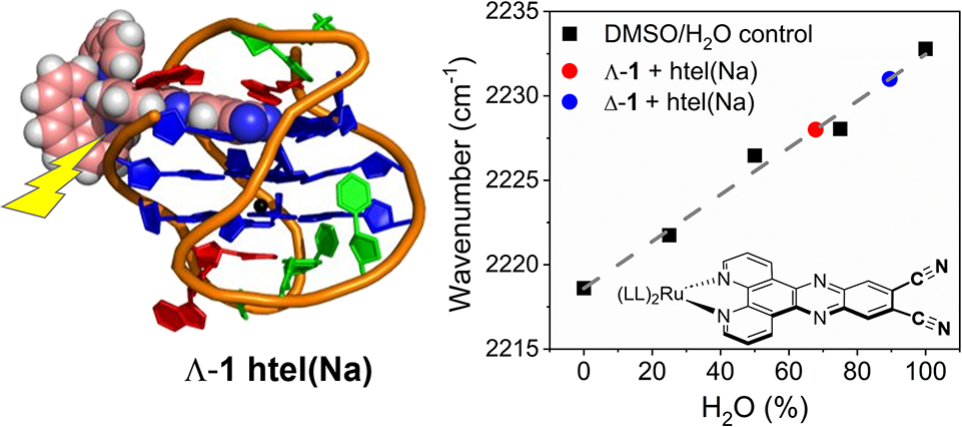

The nitrile containing Ru(II)polypyridyl complex [Ru(phen)_2_(11,12-dCN-dppz)]^2+^ (**1**) is shown to act as a sensitive infrared probe of G-quadruplex (G4) structures. UV–visible absorption spectroscopy reveals enantiomer sensitive binding for the hybrid **htel(K)** and antiparallel **htel(Na)** G4s formed by the human telomer sequence d[AG_3_(TTAG_3_)_3_]. Time-resolved infrared (TRIR) of **1** upon 400 nm excitation indicates dominant interactions with the guanine bases in the case of Λ-**1/htel(K)**, Δ-**1/htel(K)**, and Λ-**1**/**htel(Na)** binding, whereas Δ-**1**/**htel(Na)** binding is associated with interactions with thymine and adenine bases in the loop. The intense nitrile transient at 2232 cm^–1^ undergoes a linear shift to lower frequency as the solution hydrogen bonding environment decreases in DMSO/water mixtures. This shift is used as a sensitive reporter of the nitrile environment within the binding pocket. The lifetime of **1** in D_2_O (*ca*. 100 ps) is found to increase upon DNA binding, and monitoring of the nitrile and ligand transients as well as the diagnostic DNA bleach bands shows that this increase is related to greater protection from the solvent environment. Molecular dynamics simulations together with binding energy calculations identify the most favorable binding site for each system, which are in excellent agreement with the observed TRIR solution study. This study shows the power of combining the environmental sensitivity of an infrared (IR) probe in its excited state with the TRIR DNA “site effect” to gain important information about the binding site of photoactive agents and points to the potential of such amplified IR probes as sensitive reporters of biological environments.

## Introduction

Our understanding of the biological function of different nucleic acid structures is still emerging.^1−3^ Notably, experimental data increasingly support the importance of G-quadruplex (G4) structures in living cells and in disease processes.^4−8^ The four stranded G4 structure is characterized by stacks of Hoogsten hydrogen-bonded guanine quartets, with a central metal ion channel, with distinctive grooves formed by lateral and end loops.^7,9−11^ The human telomer sequence (**htel**) can adopt a variety of structures (parallel, antiparallel and hybrid folded) depending on the metal ion (Na^+^/K^+^), with the parallel K^+^ structure generally favored *in vivo*.^7^ It is predicted that there are over 700,000 G4 forming sequences in the human genome.^12^ Importantly, G4 forming sequences are overrepresented in oncogenes,^5,7,13,14^ and elevated levels of G4 formation have been detected in human cancer tissue taken from the stomach, liver^15^ and breast.^16^ Most notably, the extension of the telomere sequence by the enzyme telomerase, which is present in 80–85% of cancer cells, is linked with cancer cell immortality.^7^ G4-based therapeutic strategies generally attempt to inhibit telomerase by stabilizing the G4 structure, hence hampering the immortality phenotype of cancer cells, or to stabilize G4-folding in oncogenes as a means to downregulate its expression. Consequently, anticancer drugs that target G4 are actively being pursued with a number in clinical trials approved,^7,17^ while there is also interest in developing diagnostic molecular probes to recognize these structures.^18^ The diversity of oncogene targets is further evidenced by their ability to form coupled ternary structures.^19,20^ While, recent studies have revealed the presence of quadruplex forming sequences in Gram-negative bacteria suggesting a possible avenue to develop G4 targeting antibiotics to tackle antimicrobial resistance.^21^ Additionally, the presence of G4 structures in viral genomes, including RNA viruses and SARS-CoV-2 makes, further highlights its potential as a therapeutic target.^22,23^

The excellent photophysical and electrochemical properties of transition metal polypyridyl complexes make them attractive candidates for DNA targeting, imaging, and phototherapeutic applications.^24−28^ In particular, their chiral octahedral geometry and modular architecture offer a unique platform to develop molecular probes to target G-quadruplex structures through concomitant binding of the base-stacks, grooves and loops.^29,30^ The first example of G4 binding by a ruthenium polypyridyl complex was for a diazo linked Ru(bpy)_2_ dinuclear system reported by the Thomas group.^31^ This report was followed by related dinuclear systems, which become emissive upon G4 binding,^32−35^ including flexible linker dinuclear systems.^36^ Notably, G4 targeting mononuclear ruthenium polypyridyl systems have been found to inhibit telomerase,^37^ to bind cellular G4 structures,^38^ to cause apoptosis in cancer cells through G4 stabilization,^39^ and to sensitize photoreaction with G4.^40^ In 2010 the “light-switch” *rac-*[Ru(bpy)_2_dppz]^2+^ (dppz = dipyridophenazine) was shown to bind the human telomeric G4 structure^41^ and improved G4 binding has been observed for substituted dppz ligand systems through end stacking of the extended dppz ligand with guanine tetrads.^42−44^ In the development of *in vitro* probes, Monchaud recently reported G4 detection by luminescent racemic complexes prepared using extended ligand systems.^45^ Notably, enantiospecific binding of hTel G4 by a ruthenium polypyridyl complex has been linked to the downstream inhibition of replication.^46^

In addition to structural recognition, the intense charge-transfer (CT) character of transition metal polypyridyl complexes can be exploited to report on diverse nucleic acid structures through the light-switch effect,^47−51^ and to trigger DNA photo-oxidation through triplet sensitized type 1 and type 2 singlet oxygen generation^52^ or through direct oxidation of guanine by photoinduced single electron transfer to the metal complex in the excited state.^53^ We are interested in developing metal polypyridyl probes that combine these diagnostic and photodamaging properties. In particular, we have used time-resolved infrared (TRIR) spectroscopy to monitor DNA photo-oxidation of guanine in diverse DNA systems in solution and in crystals^53−56^ and recently reported the photo-oxidation of adenine by a chromium polypyridyl complex.^57^ We have extensively used the “site effect” to report on the site of photo-oxidation.^53−56^ This effect leads to diagnostic bleach bands due to the perturbation of the nucleobases in the binding site of light activated metal polypyridyl probes and has been used to distinguish loop interactions from G-quartet stacking interactions for the *rac*-[Ru(phen)_2_dppz]^2+^ light switch complex bound to different structures of the human telomer sequence (**htel**) in solution.^58^ In a related study we identified the role of cytosine stacking interactions in i-motif DNA,^59^ and very recently used TRIR in combination with NMR to resolve the solution binding of a light switch NIR osmium dppz complex to c-myc and **htel** G4 structures.^60^

**Figure 1:**
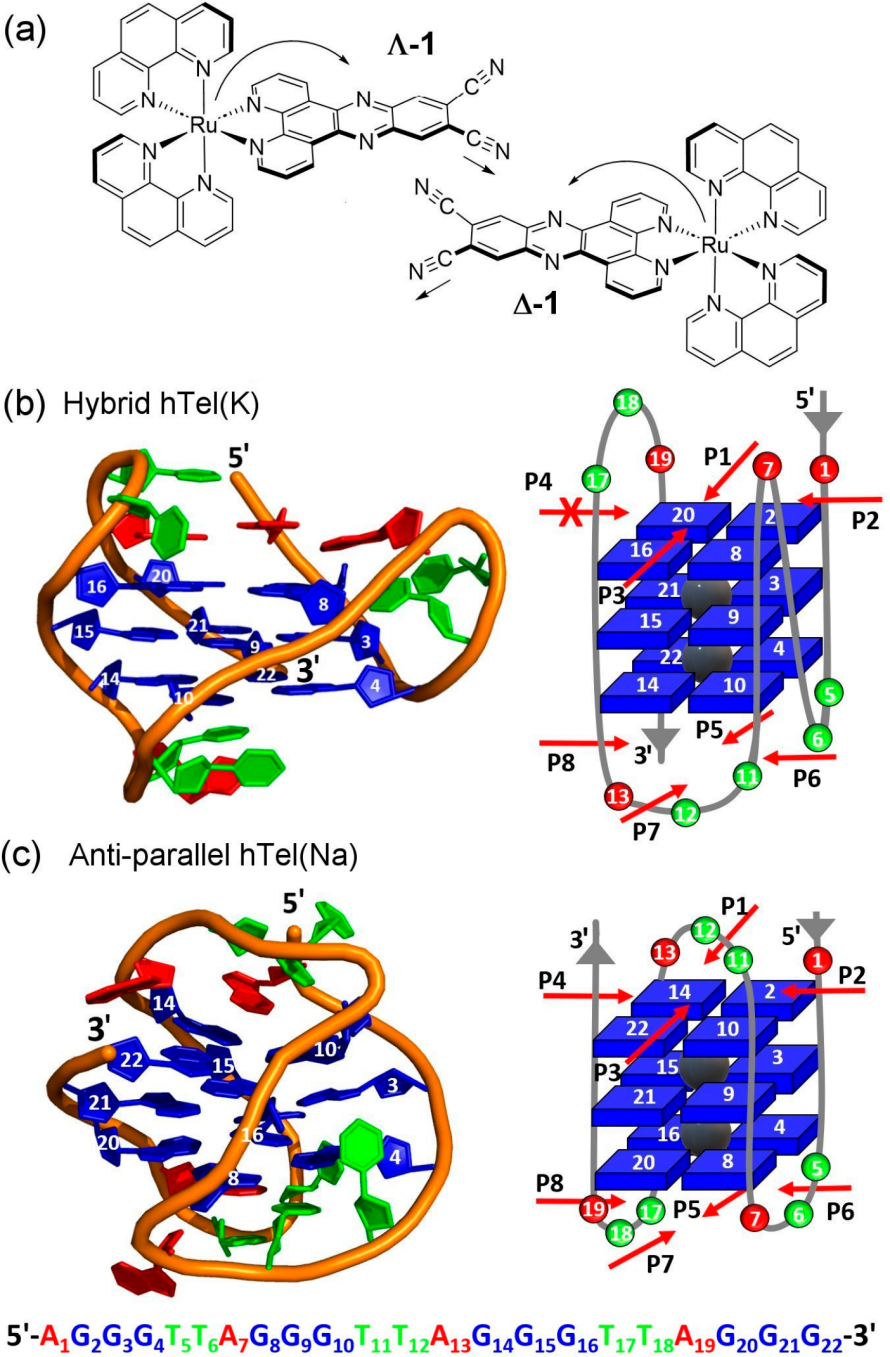
(a) Structure of (a) Δ-**1** and (b) Λ-**1** highlighting the dppz based ^3^MLCT (Metal to Ligand Charge Transfer) excited state and the nitrile vibrations. (b) G4 structures formed from the human telomere sequence d[AG_3_(TTAG_3_)_3_] in the presence of potassium cations hybrid **htel(K)** PDB: 2HY9(76) and sodium cations antiparallel **htel(Na)** PDB: 143D.^77^ Arrows indicate the end on binding approaches of **1** to the upper and lower G4 tetrads.

In this study we highlight the diagnostic ability of the ruthenium polypyridyl complex [Ru(phen)_2_(11,12-dCN-dppz)]^2+^ (**1**) that contains a nitrile (CN) infrared (IR) probe (see Figure 1). IR probes are powerful molecular tools that, through the phenomenon known as the Vibrational Stark Effect (VSE), have been used to monitor the local environment in chemically important processes.^61,62^ The nitrile stretching vibration is sensitive to its local electrostatic environment, and it is possible to exploit the fact that different solvents exert different “effective” electric fields on the probe, which result in a shift in the position of the frequency of the nitrile band.^63,64^ Nitrile probes are also sensitive to the hydrogen bonding (HB) environment accessed in DMSO/water mixtures.^65^ The sensitivity of the nitrile vibration to changes in the electrostatic field, together with the location of its vibration in a distinct IR window, has made it a particularly attractive probe to characterize complex molecular environments,^66,67^ and this has been used to characterize proteins,^68^ conformational changes in sensory Rhodopsin II,^69,70^ enzyme active sites,^71^ lipid membranes,^72^ and nucleic acids.^73^ Nitrile probes have also been used to quantify the effect of changing electric fields on the enzyme active site by monitoring the magnitude of the electrostatic perturbation introduced by photoexcitation of a fluorescent analogue of the reaction intermediate.^63^ Complex **1** was chosen as a model complex due to the symmetric substitution of nitrile groups at the terminal carbons on the intercalating dppz ligand. X-ray crystallographic studies have suggested that substitution of nitrile groups on the extended dppz ligand improves intercalation by stabilizing nucleobase interactions with double-stranded DNA.^74^ Related structural studies on the binding of a tetraazaphenanthrene (TAP) containing complex [Ru(TAP)_2_(11-CN-dppz)]^2+^ with quadruplex DNA showed favorable stacking interaction between the 11-CN-dppz ligand and the guanine bases complemented by polar contacts between the nitrile substituent and a 2-NH_2_ on guanine.^75^

In the combined spectroscopic and molecular dynamics (MD) study below, we resolve the binding interactions of the Λ-**1** and Δ-**1** enantiomers with the hybrid type **htel(K)** and antiparallel **htel(Na)** G4 structures formed by the human telomer sequence d[AG_3_(TTAG_3_)_3_]. These structures possess distinct loop arrangements with two lower lateral loops and one upper diagonal loop in **htel(Na)** and two lateral loops and one side propeller loop in **htel(K)**. Significantly, our TRIR study shows that the intense nitrile transient formed upon excitation of **1** can act as a sensitive probe of the solution HB environment. We use this response, combined with (i) the associated excited state lifetime and (ii) the identity of the bases in the binding site determined using the “site effect”, to gain information on the environment within the G4 binding site in solution. To identify the likely site of interaction, MD simulations are used to examine the binding to the terminal G4 tetrads, which can be achieved through eight possible binding approaches, four to the upper (**P1–P4**) and four to the lower G4 tetrad (**P5–P8**) (see Figure 1). Together, the TRIR results supported by MD simulations provide an exquisite picture of the landscape of photoactivated probes, which can now be applied to the development of phototherapeutics and diagnostics.

## Results

The UV–visible electronic absorption spectra of the chloride salt of **1**^2+^ in aqueous solution show intense transitions observed between 250 and 300 nm assigned to singlet ligand-centered (^1^LC) excitations associated with the polypyridyl ligands.^78^ The broad absorption envelope between 370 and 520 nm shows a distinctive dppz LC n-π* transition (380 nm) and a singlet MLCT transition (^1^MLCT, d-π*).^78^ The complex is found to be nonemissive in water, which is also the case for the parent [Ru(phen)_2_dppz].Cl_2_ complex.^50^ The circular dichroism (CD) spectra for the Δ and Λ stereoisomers of [**1**^2+^][Cl]_2_ show opposite (but equal) Cotton effects with characteristic couplets observed for the ^1^LC and ^1^MLCT transitions (see Figure S1).

### Visible Absorption Quadruplex Binding Studies

**Figure 2:**
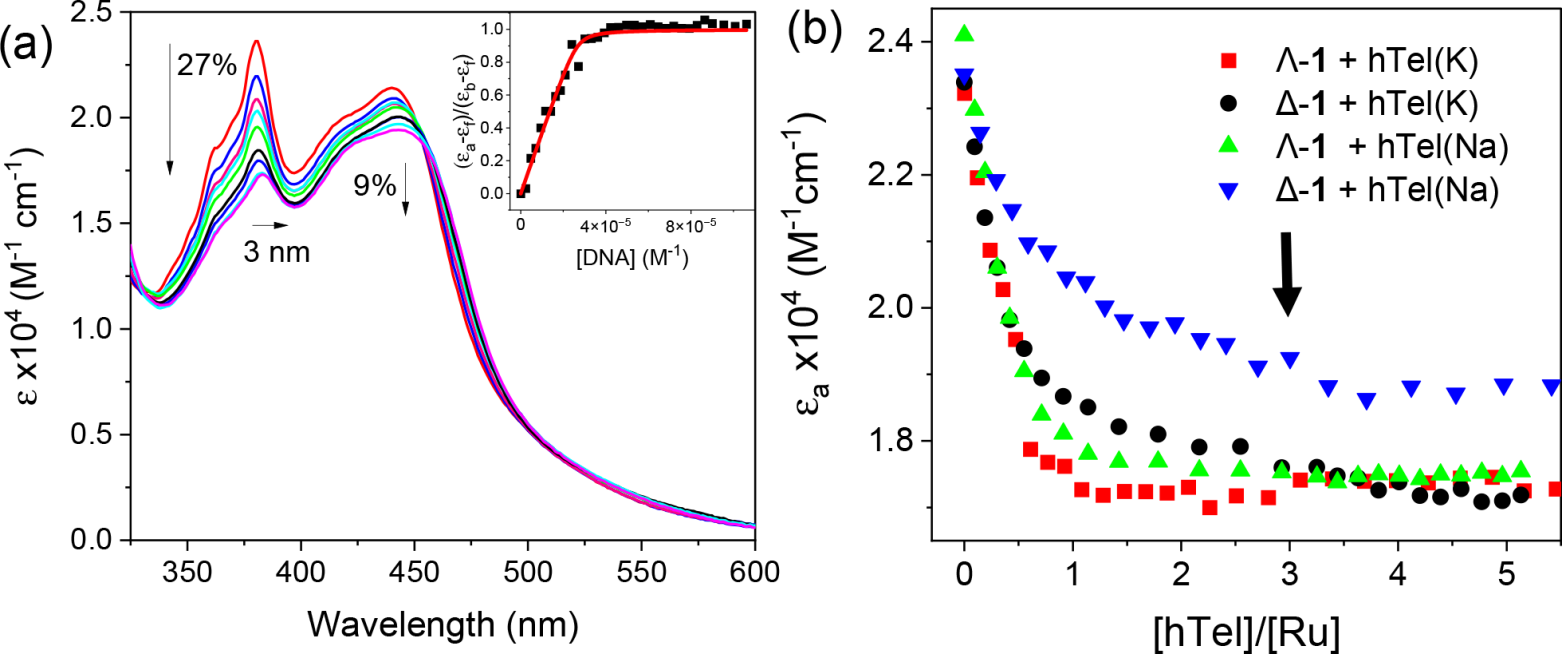
(a) UV–visible absorbance spectra of Λ-**1** (7.2 μM) titrated against increasing concentration of **htel(K)** (0→ 37 μM) in 50 mM phosphate buffer and 100 mM KCl at pH 7. (b) Comparison of changes in absorbance at (380 nm) for the quadruplex systems. Arrow indicates the effective binding that has occurred upon addition of three equivalents of the **htel(K)** structure.

Visible absorption titrations were performed to study the affinity of Δ-**1** and Λ-**1** to the hybrid **htel(K)** and antiparallel **htel(Na)** structures shown in Figure 1. In all cases the addition of increasing quadruplex DNA resulted in pronounced hypochromism at the 380 nm band associated with the LC of the dppz ligand (ca. 30% reduction in intensity) accompanied by a slight red shift ∼3 nm, with weaker hypochromism (*ca*. 16% reduction) in the ^1^MLCT transition at 440 nm (see Figure 2a and Figure S2). These observations are characteristic of the close association of the dppz ligand with DNA.^53^ In all cases, these significant hypochromism changes are observed to reached a plateau upon the addition of 3 equiv of G4 (Figure 2b). For both structures the Λ-enantiomer was found to undergo sharper hypochromism changes which suggests a more defined binding site. The DNA binding constants (*K*_b_) and binding site size (per G-tetrad) for Δ-**1** and Λ-**1** were determined by fitting the changes at 380 nm to a modified binding model developed by Bard et al.^79^ The complexes were found to have varying affinity for the quadruplex structures with *K*_b_ values ranging from 10^5^ M^–1^ to 10^7^ M^–1^ (see Figure S3 and Table 1). The highest affinity was observed for the binding of Λ-**1** to **htel(K)** with *K*_b_ = (1.1 ± 0.6) × 10^7^ M^–1^, and the weakest affinity was observed for the binding of Δ-**1** to **htel(Na)** with *K*_b_ = (1.9 ± 0.7) × 10^5^ M^–1^. A binding site size of 1.8 G-tetrads per complex was estimated for Λ-**1/htel(K)**, while a smaller site size of 1.1 G-tetrads was estimated for the binding of Δ-**1** to **htel(Na)**. It should be noted that, in common with other dppz containing Ru(II)polypyridyl complexes,^75^ the enantiomers of **1** were also found to bind to double stranded (dsDNA) (see Figure S4). Greater affinity was observed for Λ-**1** with *K*_b_ = (1.4 ± 0.3) × 10^6^ M^–1^ compared to Δ-**1** to (3.5 ± 0.9) × 10^5^ M^–1^ for Δ-**1**. These results indicate that Λ-**1** has a preference for the **htel(K)** over dsDNA.

**Table 1:** Bard Binding Affinity Calculated for Changes in Absorbance at 380 nm in Terms of [G-tetrad]

DNA	Binding constant	Binding site size (per G-tetrad)	*R*^2^
Λ-**1** + htel(K)	1.1(±0.6) × 10^7^ M^–1^	1.8(±0.1)	0.982
Δ-**1** + htel(K)	4.2(±0.5) × 10^5^ M^–1^	1.5(±0.1)	0.978
Λ-**1** + htel(Na)	2.2(±0.3) × 10^6^ M^–1^	1.5(±0.1)	0.996
Δ-**1** + htel(Na)	1.9(±0.7) × 10^5^ M^–1^	1.1(±0.3)	0.995

### Time-Resolved Infrared Studies

**Figure 3:**
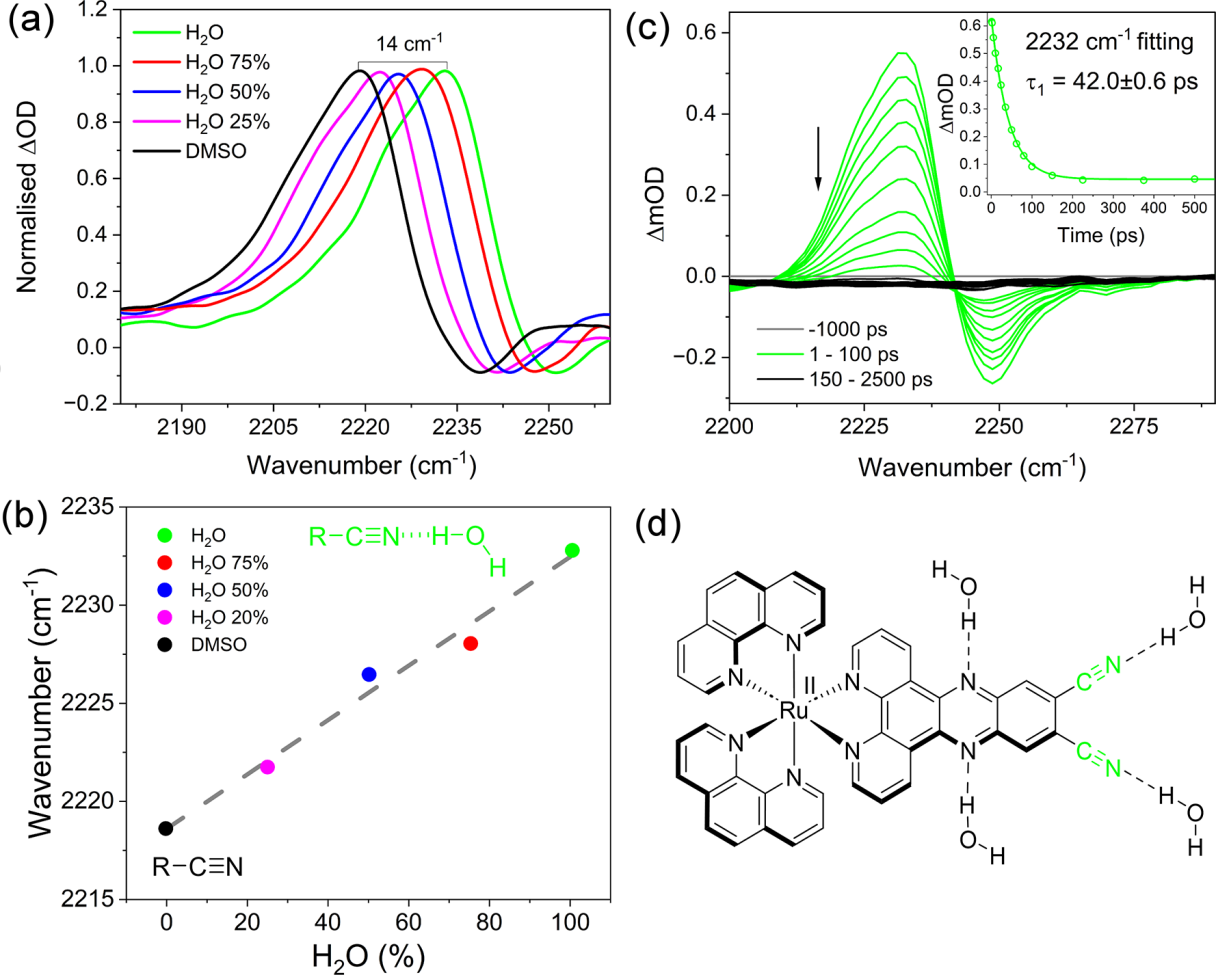
Solvent dependent nitrile response (a) TRIR spectra of 1 mM *rac*-**1** recorded at 10 ps in different solution compositions of DMSO and water recorded after excitation, (λ_exc_ = 400 nm). Correlation of the position of the transient band with the HB environment of the surrounding solvent. (c) Kinetic analysis of the nitrile transient. (d) Depiction of the HB interactions of **1**.

The ground state IR (FTIR) spectrum of the racemic (*rac*-**1**) complex recorded in D_2_O shows the presence of several bands below 1500 cm^–1^ arising from vibrations on the phen and 11,12-dCN-dppz ligands, (Figure S1). Recording the spectrum in H_2_O reveals the characteristic nitrile vibration at 2251 cm^–1^. Below 1550 cm^–1^, the TRIR spectra reports on the behavior of the complex of *rac*-**1** in D_2_O excited at 400 nm and shows a series of transient bands and weak bleaches assigned to vibrational modes on the phen and the phenazine section of the polypyridyl ligands (Figure S5).^1^ The TRIR spectrum of **1** between 1550 and 1675 cm^–1^, which overlaps with the region where DNA base vibrations are detected, is comparatively quiet. Well separated from these regions is the nitrile window (2100–2300 cm^–1^). Due to the strong absorbance of D_2_O in this region of the spectrum, the TRIR spectra of *rac*-**1** were recorded in H_2_O and show a transient at 2232 cm^–1^ whose intensity is significantly greater than the associated bleach band (see Figure 3a). The notable enhancement of the molar absorptivity of the ν(C≡N) vibration in the excited state is in agreement with recent observations for indole substituted nitriles and linked to the electron density in the aromatic system.^80^ The transient band is shifted to a lower wavenumber by *ca*. 18 cm^–1^ from the position of the bleach ground state absorption, indicating the presence of a weaker CN bond in the ^3^MLCT* excited state relative to the ground state. The downshift of the ν(C≡N) has been previously observed for ruthenium polypyridyl complexes and is attributed to the population of a ligand based π* antibonding orbital, which weakens the ν(C≡N) bonds.^81,82^

The C≡N stretching vibration is known to be sensitive to the HB environment.^63,65,67,83^ As the HB environment of **1** is expected to decrease upon DNA binding, the nitrile transient was recorded in different protic environments by increasing the water content in a DMSO/water mixture from 0% to 100% in 25%(v/v) increments (see Figure 3a). The position of the nitrile transient band ν(C≡N) was observed to undergo a linear shift to a higher wavenumber from 2219 cm^–1^ in 100% DMSO to 2233 cm^–1^ in 100% H_2_O (Figure 3b), which tracked the shift in the ground state bleach from 2240 cm^–1^ in 100% DMSO to 2251 cm^–1^ in 100% H_2_O (Table S2). This trend is similar to what has been observed for phenyl nitrile as a function of the hydrogen bonding environment^67^ and in DMSO/H_2_O mixtures.^65^

**Figure 4:**
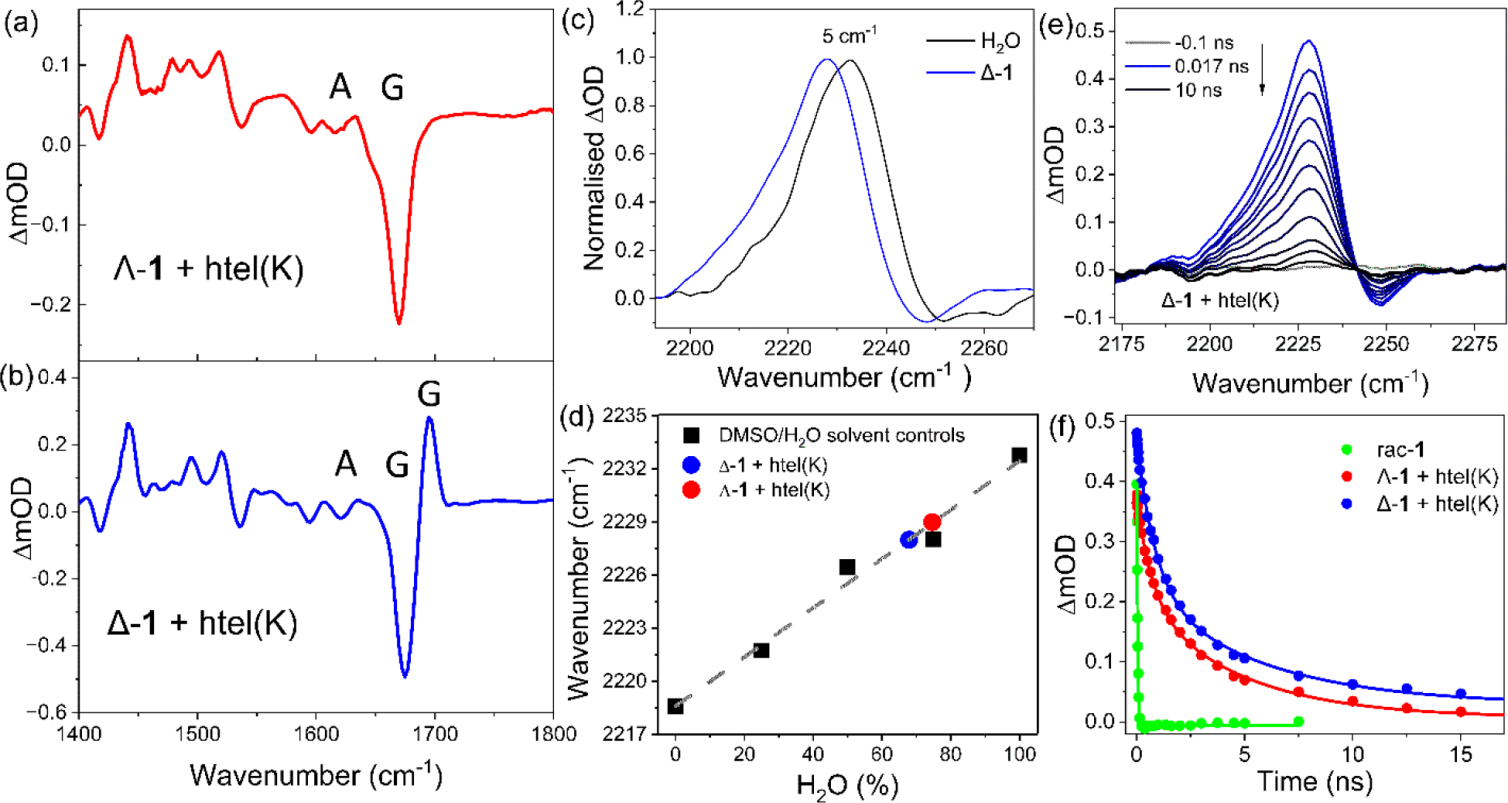
TRIR spectra of 0.4 mM (a) Λ-**1** (red line) and (b) Δ-**1** (blue line) in the presence of 1.2 mM **htel(K)** in 50 mM potassium phosphate buffer and 100 mM KCl in D_2_O, (c) shift in the Λ-**1** nitrile transient in the presence of **htel(K)** recorded at 17 ps. (d) Correlation of the position of nitrile transient for G4-bound Λ-**1** and Δ-**1** to the HB nature of the solution environment. (e). TRIR spectra of the nitrile of Λ-**1** bound to **htel(K)** recorded between 17 ps and 10 ns. (f) Comparative kinetics for **1** in aqueous solution and bound to **htel(K)**.

The TRIR spectra show that the excited state of **1** in aqueous solution undergoes rapid decay to the ground state with no evidence of vibrational cooling observed at early times (1–10 ps). The transient bands at 1347 and 1440 cm^–1^ recorded in D_2_O decay with an average lifetime of 100 ps obtained by monoexponential fitting, while faster decay was observed ca. 50 ps when the measurement is performed in H_2_O (Figure S5). The nitrile transient band at 2232 cm^–1^ in H_2_O was also observed to undergo complete decay with concomitant recovery of the bleach band on a faster time scale (by first order kinetics τ = 42.0 ± 0.6 ps) (see Figure 3c). The difference in the kinetics between D_2_O and H_2_O has also been observed for the related [Ru(phen)_2_dppz]Cl_2_ light switch complex, which is nonemissive in water and is attributed to the impact of the solvent interactions on the excited state, Figure 4d.^48^ Additionally, the TRIR spectra of **1** recorded for the nitrile window at 10 ps, 100 ps, and 1 ns time delays in pure DMSO and DMSO/water mixtures reveal that the excited state becomes longer-lived in the more aprotic environment (Figure S6).

### TRIR Studies of G4 Bound Systems

Next, TRIR was used to characterize Λ-**1** and Δ-**1** when bound to the two G4 structures. The experiments were performed under conditions where **1** is fully bound, which was determined from the visible absorption titration (Figure 2) to be at a ratio of 1:3 complex:G4. The presence of excess G4 structures also minimized multiple interactions of **1** with an individual G4 structure. The TRIR spectrum recorded 17 ps after 400 nm excitation of Λ-**1** in the presence of hybrid **htel(K)** yields a structured spectrum between 1300 and 1750 cm^–1^ (Figure 4a and Figure S7a). A sharp bleach is observed at 1670 cm^–1^, which is characteristic of the guanine carbonyl and arises due to the perturbation of the ground state vibration due to the close proximity of the excited state (see Figure 4a).^58^ A weak transient band is observed at 1690 cm^–1^, which is also associated with the perturbation of guanine.^84^ A similar spectrum is obtained for Δ-**1**; however, an additional transient is observed at 1690 cm^–1^, which overlaps with the bleach band. This new absorbance feature is attributed to a shift of a guanine carbonyl vibration to a higher wavenumber due to the presence of the excited state, which may arise due to a polar interaction between the guanine base and the nitrile group (Figure 4b and Figure S7b). The absence of strong bleaches associated with adenine (*ca*. 1620 cm^–1^) and thymine (*ca*. 1640 cm^–1^, 1662 cm^–1^, 1705 cm^–1^)^84^ suggests that the hybrid **htel(K)** binding pocket of Δ-**1** is largely associated with guanine interactions. Additionally, when bound to **htel(K)**, the nitrile transient of Δ-**1** is observed to shift 5 cm^–1^ from 2233 cm^–1^ in H_2_O to 2228 cm^–1^ (Figure 4c) with a 4 cm^–1^ shift observed for Λ-**1** (to 2229 cm^–1^). This shift in the nitrile transient band for Λ-**1** and Δ-**1** indicates that the binding site environment for both enantiomers has reduced access to HB compared to when **1** is free in solution. This effect is slightly more pronounced for Δ-**1**, suggesting that it is bound in a more hydrophobic environment than Λ-**1** (Figure 4d), which also results in different interactions with the guanine bases leading to the transient at 1690 cm^–1^. The TRIR spectra of the nitrile band recorded at early time 1–10 ps showed very little evidence of vibrational cooling (see Figure S8).

Kinetic analysis of the nitrile transient revealed a significant increase in the lifetime of Δ-**1** (τ_1_ = 840 ± 80 ps and τ_2_ = 5.5 ± 0.8 ns) and Λ-**1** (τ_1_ = 710 ± 90 ps and τ_2_ = 4.6 ± 0.5 ns) upon binding to the hybrid **htel(K)** (see Figure 4e–f, Figure S9 and Table 2). Similar results were obtained by fitting either the DNA bleach at 1670 cm^–1^ or the complex transient at 1347 cm^–1^ (see Table 2). On average, the increase in lifetime was found to be comparable for both enantiomers with a slightly longer lifetime observed for Δ-**1**, which is attributed to its protection from the solution environment.

**Table 2:** Summary of the Kinetic Analysis Obtained from the TRIR Spectra for Δ-**1** and Λ-**1** in the Presence of the G4 Systems

	DNA bleach (D_2_O)		Nitrile transient (H_2_O)	
System	τ_1_ (ps)	τ_2_ (ns)	τ_1_ (ps)	τ_2_ (ns)
***rac*****-1**	91 ± 5	–	40 ± 1	–
**Δ-1/htel(K)**	930 ± 280 (28%)	4.8 ± 0.7 (72%)	840 ± 80 (55%)	5.5 ± 0.8 (45%)
**Λ-1/htel(K)**	a1100 ± 300 (57%)	a4.4 ± 1.8 (43%)	710 ± 90 (44%)	4.6 ± 0.5 (56%)
**Δ-1/htel(Na)**	80 ± 4 (63%)	6.5 ± 0.4 (36%)	180 ± 10 (79%)	3.0 ± 0.5 (21%)
**Λ-1/htel(Na)**	77 ± 30 (28%)	6.0 ± 0.6 (72%)	370 ± 30 (54%)	4.5 ± 0.4 (46%)

a Kinetics determined from the transient associated with **Λ-1** 1347 cm^–1^.

**Figure 5:**
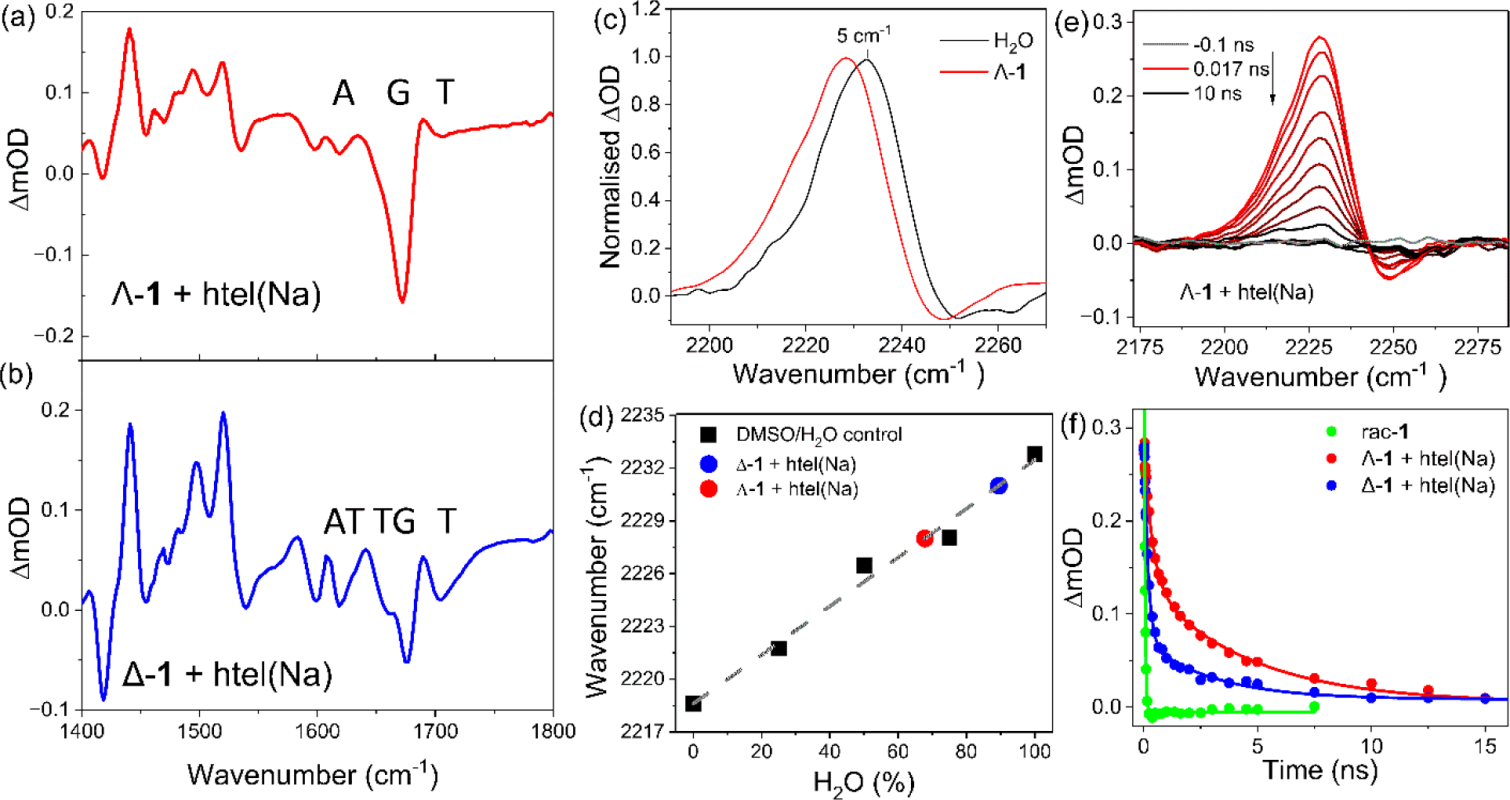
TRIR spectra of 0.4 mM (a) Λ-**1** (red line) and (b) Δ-**1** (blue line) and in the presence of 1.2 mM **htel(Na)** in 50 mM sodium phosphate buffer and 100 mM NaCl in D_2_O, (c) shift in the Λ-**1** nitrile transient in the presence of **htel(Na)** recorded at 17 ps. (d) Correlation of the position of nitrile transient for Λ-**1** and Δ-**1** bound to G4 to the HB nature of the solution environment. (e) TRIR spectra of the nitrile band recorded for Λ-**1** bound to **htel(Na)** recorded between 17 ps and 10 ns. (f) Comparative kinetics for the **1** bound to **htel(Na)**.

Having detected evidence of subtle differences in the binding site environment and kinetics for Λ-**1** and Δ-**1** bound to the hybrid **htel(K)** G4 structure attention turned to the binding interactions with the antiparallel hybrid **hel(Na)** G4 structure, for which greater sensitivity to the enantiomeric form was indicated by the visible absorption G4 titration (Figure 2b). Striking differences are observed in the TRIR spectra recorded upon 400 nm excitation of each enantiomer in the presence of **htel(Na)** (see Figure 5a–b and Figure S10). In the case of Λ-**1**, the TRIR spectrum recorded at 17 ps is dominated by the presence of a sharp bleach at *ca*. 1675 cm^–1^ characteristic of the guanine carbonyl vibration, with an additional bleach at 1620 cm^–1^ characteristic of the adenine ring vibration, and a minor contribution of thymine bleaches observed at 1660 and 1705 cm^–1^. However, a far more structured TRIR spectrum is observed for Δ-**1** in this DNA window. The bleach associated with guanine 1675 cm^–1^ is now flanked by thymine bleaches at 1660 and 1705 cm^–1^, and there is also an adenine bleach at 1620 cm^–1^ (Figure 5b). The presence of these adenine and thymine bleaches indicates that there are significant loop interactions in the binding pocket of Δ-**1** with the antiparallel **htel(Na)**, which are less prevalent in the Λ-**1**/**htel(Na)** binding site. Related to this, the shift in the position of the nitrile transient upon G4 binding is found be significantly less for Δ-**1** (2 cm^–1^) than for Λ-**1** (5 cm^–1^), which indicates that Δ-**1** is less protected from solution HB interactions compared to Λ-**1**, Figure 5c–d.

The guanine bleach recovery and the nitrile transient decay recorded for each enantiomer bound to **htel(Na)** was fitted using biexponential function and revealed clear differences in the lifetimes of the two enantiomers (Figure S11). In the case of the nitrile transient, it was found to be longer-lived for Λ-**1/htel(Na)** (τ_1_ = 370 ± 30 ps and τ_2_ = 4.5 ± 0.4 ns) compared to Δ-**1/htel(Na)** (τ_1_ = 180 ± 10 ps and τ_2_ = 3.0 ± 0.3 ns) (see Figure 5d–e and Table 2). The difference in the lifetime can be rationalized in terms of the different access of Δ-**1** to the solution environment, as evidenced by the interactions with the loops and the weak shift in the nitrile transient. Again, in contrast to the free complex, minimal changes were observed in the intensity of the nitrile band at very early times 1–10 ps (see Figure S12).

### Classical Molecular Dynamics Study of Ligand-Quadruplex Binding Modes

#### Optimization of the Quadruplex Structures

Using a successful methodology that we previously implemented to study organic probes interacting with G4 DNA,^85^ a classical molecular dynamics study was undertaken to explore the optimal binding sites for Λ-**1** and Δ-**1** with the two G4 structures. The hybrid human telomere structure resolved by Dai et al.^76^ (PDB: 2HY9) was used to model the hybrid **htel(K)** conformation by removing two additional adenine groups at the termini of the sequence, while the antiparallel conformation **htel(Na)** was modeled using the structure determined by Wang et al.^77^ (PDB: 143D). Both structures were retained after manual inclusion of central metal cations between the G-quartet stacks, which were omitted from the reported structures (Figure S13). Nanosecond long simulations were run to collect a statistically relevant number of conformers that were then used to estimate thermodynamic properties. The Gibbs free energy of binding (Δ*G*_binding_) of Λ-**1** and Δ-**1** to the G4 DNA was evaluated with the Molecular Mechanics Implicit Solvent Model Surface Area (MM-ISMSA) method^86^ by using an ensemble of conformers for each of the binding modes identified. Entropic contributions were not included but assumed to be equivalent for all of the binding modes considered.

Previous studies on the binding interactions of ruthenium polypyridyl complexes to G4 DNA have shown strong interactions through end stacking and loop regions.^23,24^ Notably, intercalation between the G-quartets has not been reported by NMR or X-ray structural studies.^33,46,75,87^ For this reason, the binding analysis was restricted to π-stacking to the upper and lower G-quartets. For each structure eight different binding approaches were considered, four to the upper G4 tetrad (**P1**-**P4**) and four to the lower (**P5**-**P8**) (see Figure 1a and 1b). The solvated geometries of the G4 bound complex were first simulated at 100 K and then gradually progressed to 300 K where the impact of the possible stacking and loop interactions associated with the binding of Λ-**1** and Δ-**1** to the binding pockets was simulated for 100 ns and constant pressure (1 atm); see methods. Using this analysis, seven potential binding positions capable of spatially incorporating Λ-**1** and Δ-**1** were identified for the **htel(K)**, with **P4** found to be too small to accommodate either enantiomer. In contrast, all eight binding approaches to the **htel(Na)** structure yielded potential binding sites (**P1**–**P8**).

#### MD Simulations of Λ-**1** and Δ-**1** to Hybrid **htel(K)**

**Figure 6:**
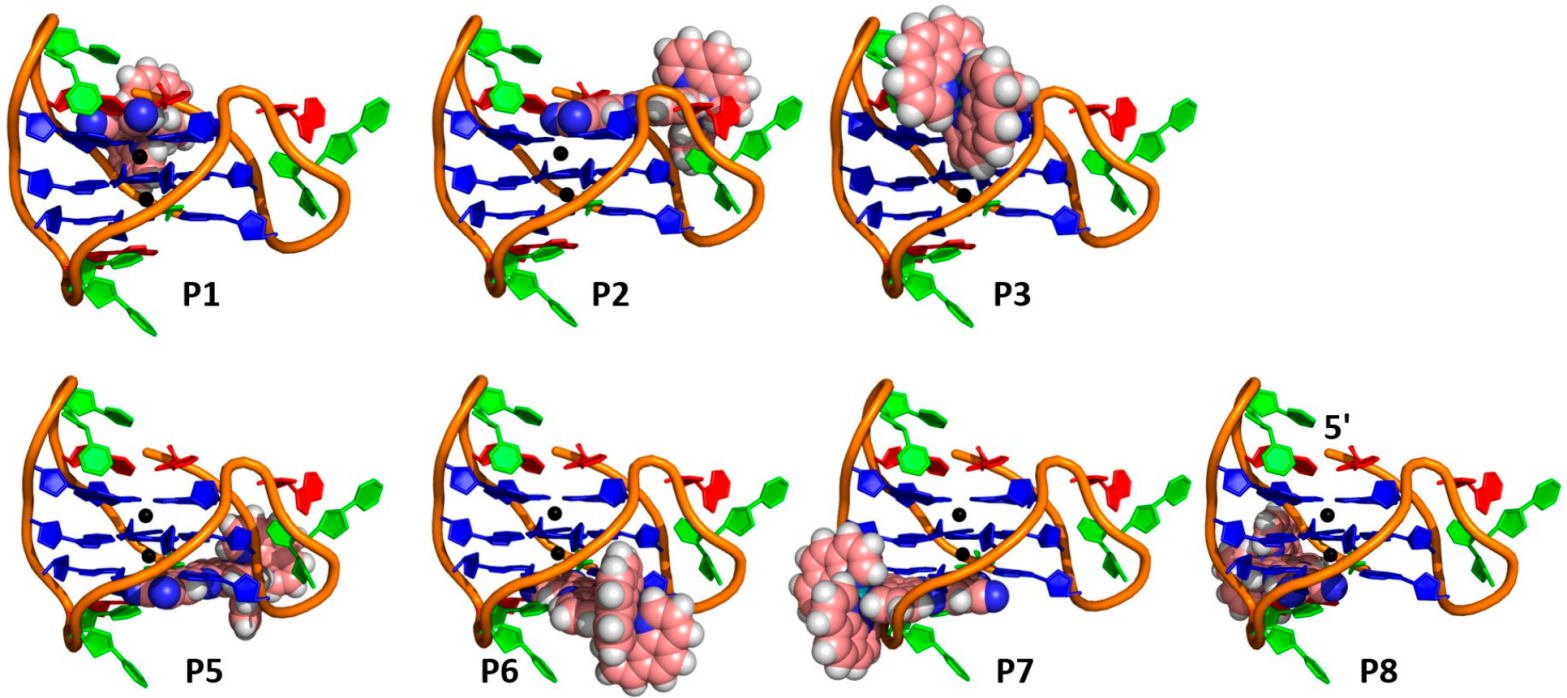
Explored human telomere sequence binding modes of a) Λ-**1** and hybrid **htel(K) (**PDB: 2HY9(76)). Guanine (blue), thymine (green), adenine (red), and potassium ions (black).

The enantiomers of **1** were manually positioned into the seven potential binding sites identified for the biologically relevant hybrid **htel(K)** conformation (see Figure 6 and Figure S14). For the Λ-**1/htel(K)** interactions the MM-ISMSA method indicates a strong interaction of Λ-**1** with several of the binding pockets with Δ*G*_binding_ in the range −65 kcal mol^–1^ to −88 kcal mol^–1^ (see Table 3 and Table S3). Interestingly, MD simulation of Λ-**1** in positions **P5** and **P8** did not produce a stable binding interaction, with the molecule leaving the binding position already while heating the system to 300 K. In the case of **P5** this is potentially due to the close interaction of Λ-**1**, with the phosphate backbone in these positions. For **P8**, this may be due to steric interactions. The most substantial interaction was observed for **P7**, where binding through the phosphate loop leads to strong interaction of the extended 11,12-dCN-dppz ligand with the lower G-quartet (see Figure 6). The weakest interactions were predicted for Λ-**1** placed at the top of the G4 structure in **P3**; these are explained by the partial inability of Λ-**1** to undergo noncovalent interactions with the nucleobases in the loop region of the hybrid conformation. The interaction of Λ-**1** in **P7** was further validated by performing an independent extended MD simulation (for 350 ns) using a randomly assigned initial velocity. This simulation produced a conformer with a comparable binding energy. This simulation highlighted the importance of nonelectrostatic interactions, in particular the stacking interactions, in the stabilization of the binding energy (Table S4). In order to analyze the stability of the DNA upon binding, we computed the root-mean-square deviation (RMSD) over the simulation time. We computed the RMSD for the whole DNA structure and the guanine bases forming the three tetrads. Notably, the simulation indicated that binding to **P7** does not cause any significant structural rearrangement of the G4 over the time period as indicated by the RMSD) with time (Figure S15). This analysis shows that Λ-**1** is very stable over the simulation length, which is to be expected due to the low energy conformation of the ruthenium octahedral geometry. Strong intramolecular Hoogsteen bonding between the guanine bases in the G-quartets, as well as the ion-dipole interactions between the guanines and central potassium cation, results in little variation in the spatial distance in time between the G-quartets in the G4 structure when Λ-**1** is located in the **P7** binding pocket (Figure S16). As expected, the most significant flexibility in the hybrid G4 conformation is observed for the loop region and is attributed to the reduced stacking and intramolecular interactions in this region. A complex that can interact in these loop regions is expected to increase the stability of this G4 conformation.

**Table 3:** Summary of the Δ*G*_binding_ Values for Λ-**1** and Δ-**1** Bound to **htel(K)** and **htel(Na)** Determined from the MM-ISMSA Study

htel(K) Position	P1	P2	P3	P4	P5	P6	P7	P8
**Δ*****G***_**binding**_ (Λ-**1**) (kcal mol^–1^)	–65.19	–70.29	–69.78	nA	nA	–68.18	**-87.87**	nA
**Δ*****G***_**binding**_ (Δ-**1**) (kcal mol^–1^)	–51.9	–61.78	–29.38	nA	–57.82	**-77.82**	–59.82	–58.56

htel(Na) Position	P1	P2	P3	P4	P5	P6	P7	P8
**Δ*****G***_**binding**_ (Λ-**1**) (kcal mol^–1^)	–62.76	–69.31	–72.74	**–94.00**	–55.64	–59.39	nA	–71.16
**Δ*****G***_**binding**_ (Δ-**1**) (kcal mol^–1^)	**–80.24**	–78.00	–51.58	–74.78	–65.19	–71.51	nA	–70.37

The MM-ISMSA study revealed weaker binding interactions (reflected in the Δ*G*_binding_) between Δ-**1** with the hybrid **htel(K)** (see Table 3 and Tables S5–S6). Again, binding to the lower G4 tetrad was found to be the most favorable, with the greatest affinity observed for Δ-**1**/**P6** with a Δ*G*_binding_ of −77.82 kcal mol^–1^. The MD simulations show that Δ-**1** is well accommodated by this pocket, and RMSD analysis shows that binding does not cause any significant conformational changes to the G4 structure (Figure S17). Indeed, Δ-**1** binding at the **P6** position appears to aid in the stabilization of the hybrid G4 conformation. The results for the **htel(K)** structure highlight the preference of the enantiomers for the different binding positions, which is readily seen for **P7**, where the Δ*G*_binding_ is significantly greater for Λ-**1** than for Δ-**1** (−87.87 kcal mol^–1^ versus −59.82 kcal mol^–1^). This reflects the impact of the different orientation of the ancillary phenanthroline ligands on the interactions with the phosphate backbone and nucleobases in the loop region, which results in a change in the angle of the 11,12-dCN-dppz stacking in the lower G-quartet. This change in angle is found to impact the VdW interaction with an energy of −91.10 kcal mol^–1^ determined for Λ-**1**/**P7** compared to −61.99 kcal mol^–1^ for Δ-**1**/**P7** (Figure S18, Table S3 and Table S5). Related to this, the simulated binding of Δ-**1**/**P3** to the top tetrad limits the interactions with the loop region, which impacts the vdW energy and results in a very low free energy of binding (Δ*G*_binding_ = −29.38 kcal mol^–1^).

**Figure 7:**
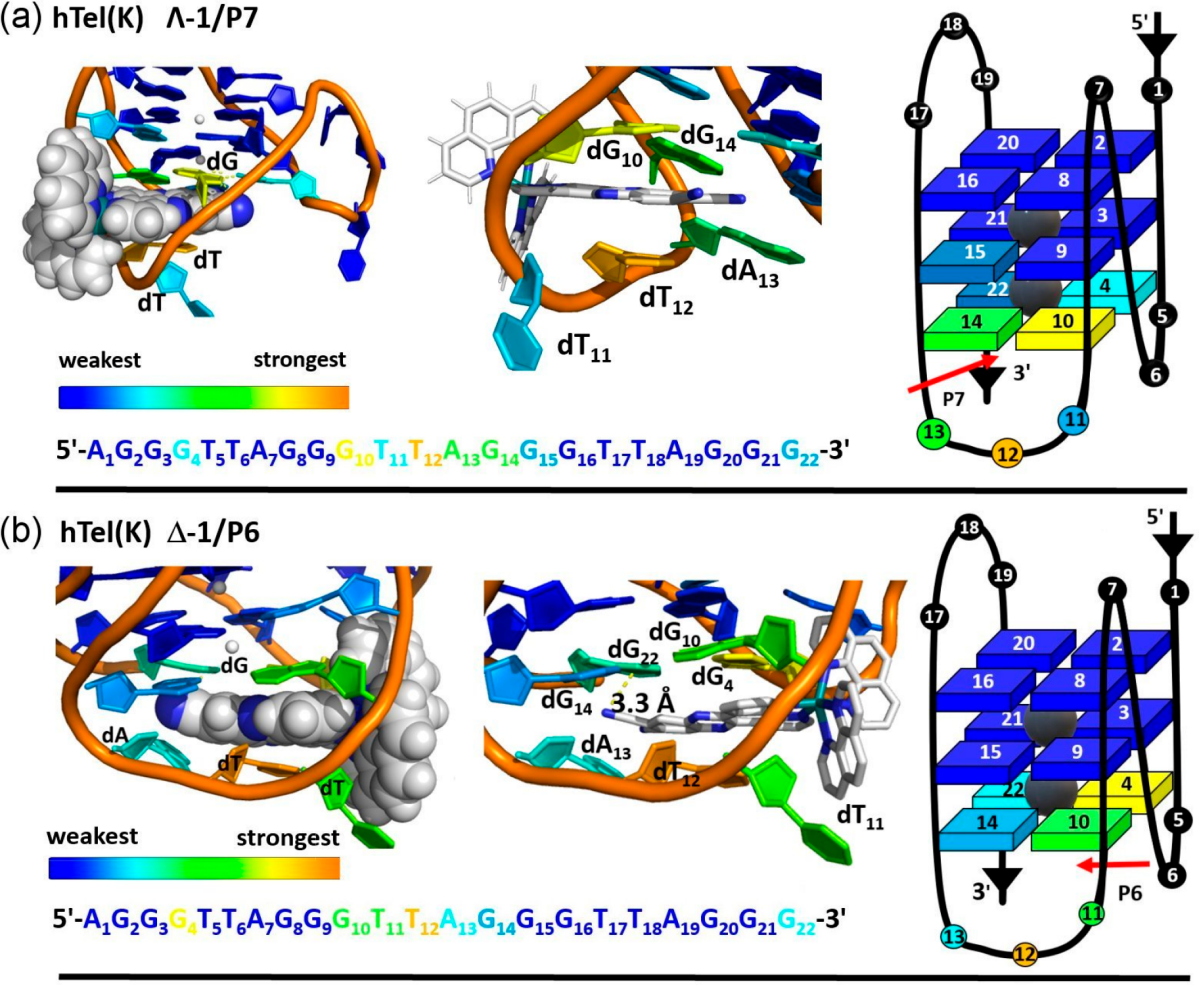
Enantiomer binding to **hel(K)** PDB: 2HY9(76) (a) Λ-**1** in **P7**. (b) Δ-**1** nucleobase interactions in **P6** and the corresponding nucleobase.

Notably, in the case of the preferred **hel(K)** binding pockets the predicted Δ*G*_binding_ is greater for Λ-**1**/**P7** than Δ-**1**/**P6**, which is in agreement with the trend in the binding constants determined from the DNA titration (see Table 2). These binding pockets were further analyzed by assessing the contribution of each single nucleobase to the Δ*G*_binding_ (see Figure 7a–b, Figure S18). In both cases, the analysis highlights significant interactions of the 11,12-dCN-dppz ligand with the four guanine bases (G_4_, G_10_, G_14_, G_20_) in the lower G-quartet. While for Λ-**1**/**P7** an additional interaction occurs between the ancillary phen ligand and G_15_ in the central G-quartet. Interactions with the adenine and thymine bases in the loops are also observed in both binding sites.

#### MD Simulations of Λ-**1** and Δ-**1** to Antiparallel **htel(Na)**

**Figure 8:**
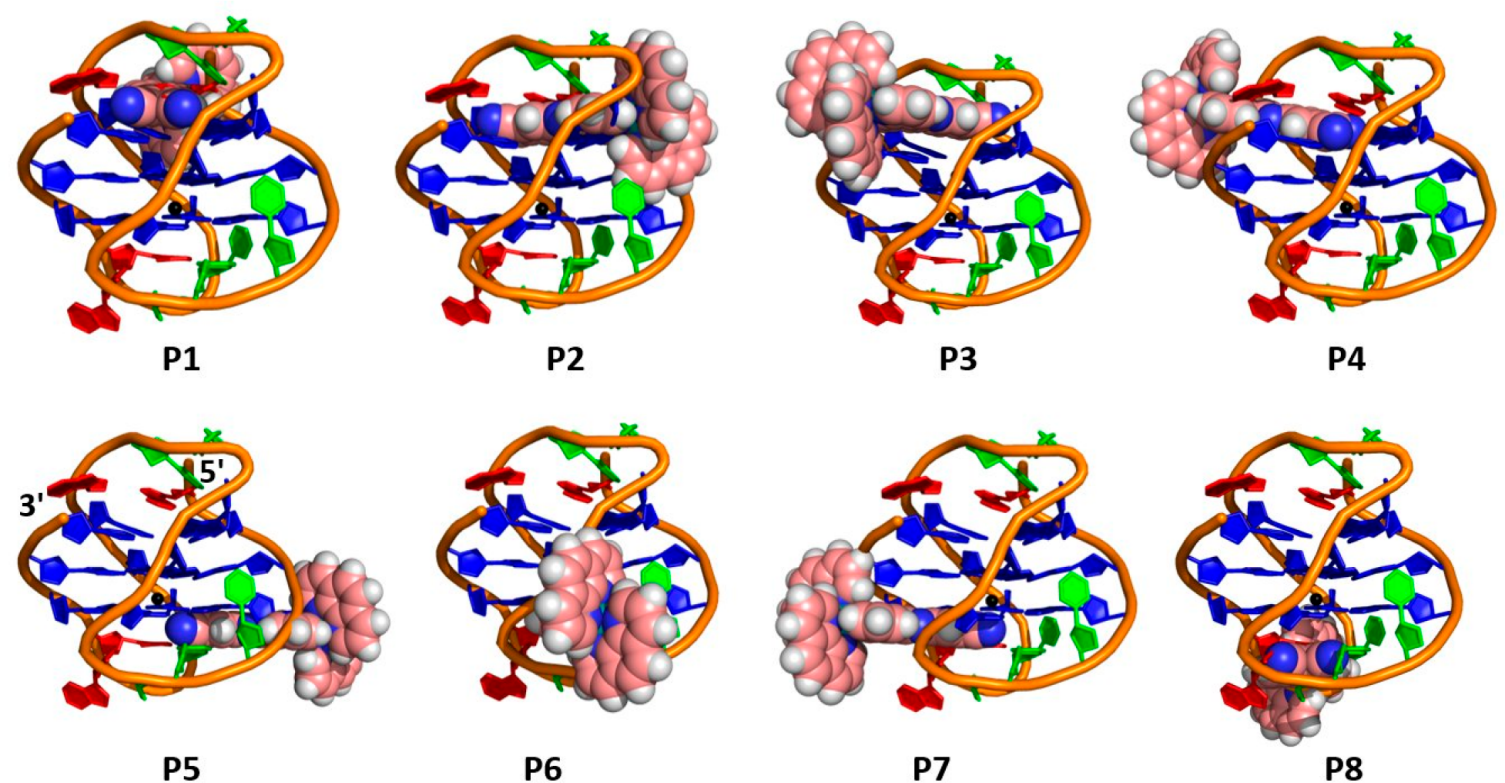
Feasible human telomere sequence binding modes of **Λ-1** and antiparallel **htel(Na)** PDB 143D. Guanine (blue), thymine (green), adenine (red), and potassium ions (black).

The MD simulated binding interactions of Λ-**1** manually positioned into the antiparallel **htel(Na)** binding sites are shown in Figure 8, and for Δ-**1** in Figure S18. Note, in these simulations binding of Λ-**1** and Δ-**1** to the lower pocket **P7** produced a nonstable binding mode, with the probe leaving the pocket during the heating phase, due to a large repulsive VdW energy. The binding site analysis for of Λ-**1** and Δ-**1** bound to the remaining sites shows clear differences in the binding interactions and affinity (Table 3 and Table S7), where in contrast to what is observed for the **htel(K)** system, a greater free energy of binding was observed for the upper G4-tetrad sites. In the case of Λ-**1** strong binding interactions are observed for all upper positions **P1–P4** and also for lower position **P8** (see Table 3). However, the greatest affinity was found for Λ-**1/P4** (Δ*G*_binding_ = −94.00 kcal mol^–1^), which is primarily due to the ability of Λ-**1** to participate in strong stacking interactions with the guanine bases in this site.

In the case of Δ-**1** the strongest interaction was found for Δ-**1/P1** (Δ*G*_binding_ = −80.24 kcal mol^–1^) (Table 3 and Table S8), which enjoyed favorable stacking interactions with the top G-quartet. Considering the VdW energy term to reflect the stacking interactions of the two enantiomers, we see that these are comparatively weaker for Δ-**1/P1** (VdW = −75.39 kcal mol^–1^) than for Λ-**1/P4** (VdW = −90.97 kcal mol^–1^). The binding analysis to the antiparallel **htel(Na)** is found to result in a more flexible interaction, which is reflected by oscillating VdW contributions to the total Gibbs binding energy observed for the two most stable positions, Λ-**1/P4** and Δ-**1/P1** (Table S9–S10).

**Figure 9:**
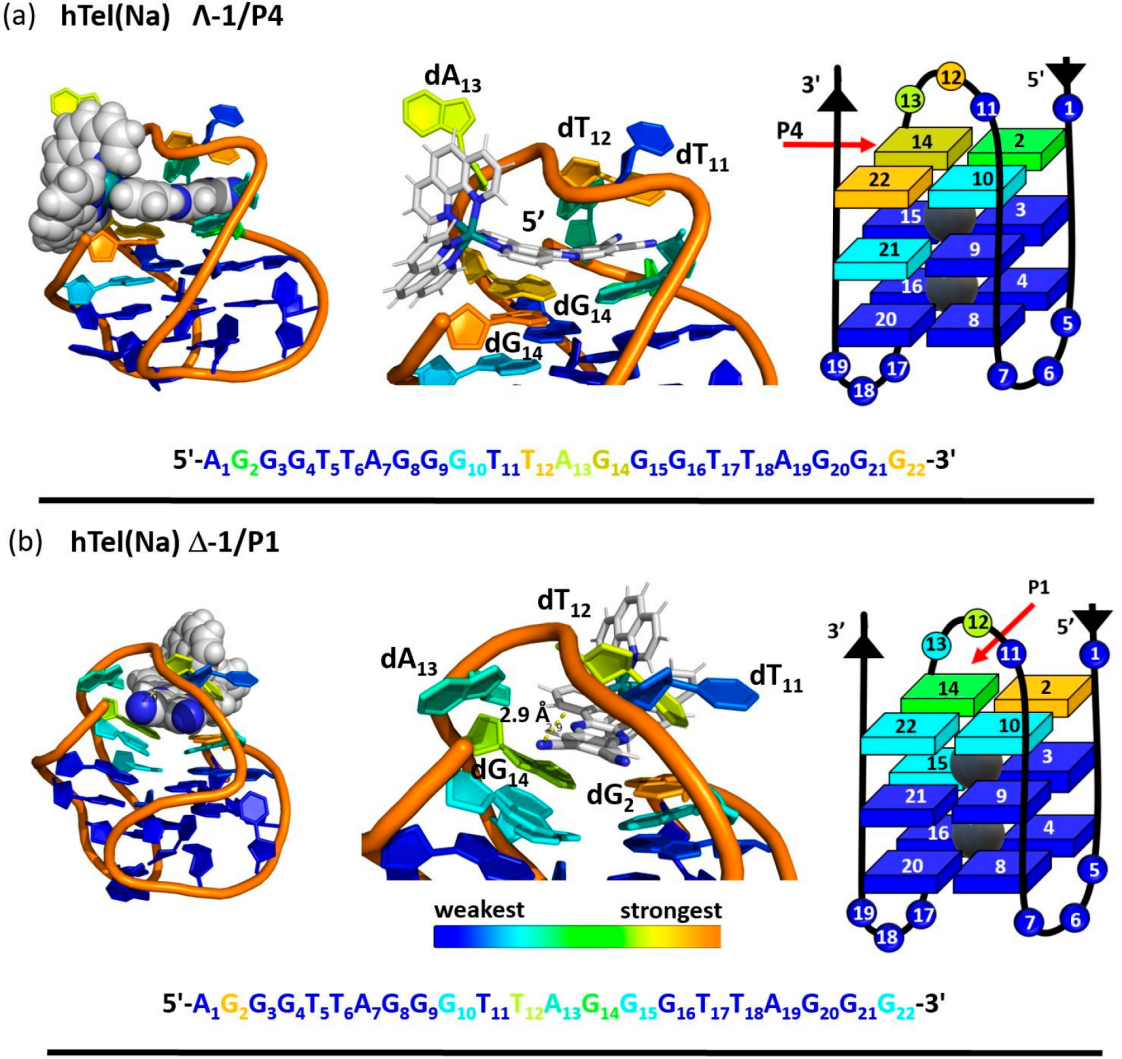
Enantiomer binding to antiparallel **hTel(Na)** PDB: 143D. (a) nucleobase interactions with Λ-**1** in **P4**, (b) Δ-**1** nucleobase interactions in **P1**.

In the case of the most stable **htel(Na)** binding sites the complex is observed to approach from adjacent sides Λ-**1**/**P4** and Δ-**1**/**P1** (see Figure 9). And the impact of this on the interactions with the individual bases was also considered. In the case of Λ-**1**/**P4** the modeling suggests good penetration of the 11,12-dCN-dppz ligand, which has good interactions with the further G_2_ base in addition to the proximal G_12_ and G_14_ bases. There are also addition guanine (G_21_) interactions with the phen ligand (see Figure 9a). The modeling also predicts that this approach results in some interactions with the adenine (A_12_) and thymine (T_13_) bases in the upper diagonal loop. These predicted interactions are very consistent with those observed in the TRIR spectrum in Figure 5a. Turning to the binding of Δ-**1** in **P1**, this approach results in less overlap with the guanine bases in the upper tetrad, and again interactions are predicted with the thymine and adenine nucleobase through the 11,12-dCN-dppz ligand. The weaker interactions with guanine are reflected in the less dominant contribution of the guanine bleach to the TRIR spectrum recorded at early time (see Figure 5b). In the case of the **htel(Na)** Λ-**1**/**P4** binding site RMSD analysis shows that binding does not cause any significant conformational changes to the G4 structure, while some fluctuation is observed in the case of Δ-**1** in the **P4** binding site across the 350 ns simulation time shown (see Figure S21).

Conformational changes to quadruplex structures can be assessed by considering fluctuations to the G-tetrad twist angle and G-tetrad separation distance.^88^ Analysis of the perturbation to these parameters upon binding of **1** was considered using the treatment described by Tsetkov et al.^88^ This analysis shows a steady separation between the two centers of mass (COMs) of the G-tetrad planes and a relatively constant twist angle for the tetrads in the case the **hel(K)** structures with greater fluctuations observed for the **htel(Na)** binding (see Figures S22 and S23). In addition, analysis of the hydrogen bond distances contributing to the Hoogsteen base pairing revealed some sensitivity to the presence of the probe over the simulation time. Here the hydrogen bond was deemed defined if the distance between the two centers was up to 3 Å and forming an angle of 20°. Across the simulations for the four systems, the middle tetrad appeared to be the most perturbed by the presence of the probe, with only a few HBs that are stable along the simulation. Overall, the HB analysis seems to confirm the trend of a stronger flexibility of the DNA in the presence of Na^+^ with respect to K^+^ (see Tables S11–S14).

Finally, additional extended simulations were run to validate the most stable binding mode and check the stability over a longer time; the calculated binding energies are reported in the Supporting Information (Tables S4, S6, S9, and S10). Each trajectory was initialized with different initial velocities and propagated for 350 ns, for a total of 1.1 μs dynamics for each of the four most stable binding positions. Notably, for all replica the systems are found to be stable, and the complex remains in the binding site. Some differences in the total binding energy are found in the different replicas, because the energy is influenced by the electrostatic energy contributions, solvation and the fluctuations of VdW interactions. The structures represent possible rearrangements of **1** in the binding pocket with different interaction energies. Yet, while possible rearrangements may occur, no interconversion with other positions was found. Highly consistent replicas were observed for binding to the **hTel(K)**, with the most consistent values observed for Δ-**1**/**P6** binding, which shows an overall stability of this binding mode that is arrived at regardless of the randomized initial conditions of the dynamics. Interestingly, TRIR suggests a significant perturbation of guanine for this site. While in the case of Λ-**1**/**P7** the small difference in the electrostatic and VdW interactions may represent a slightly different overlap in the pocket. However, in the case of the **htel(Na)** structures Δ-**1**/**P4** and Δ-**1**/**P1** binding, further sampling yields a more diverse number of arrangements. These results reveal that in addition to configuration with a lowest energy value other binding interactions are available in the binding pocket. Overall, the modeling for the G4 binding of Λ-**1** and Δ-**1** underlines the importance of multiple noncovalent interactions in the loop and tetrad regions of the structure in directing the preferred binding site.

## Discussion

The distinctive shape and size of the grooves and loops of G4 structures offer the potential to develop molecules whose shape are tailored to target a specific structure.^89^ The chiral nature and versatile structure of ruthenium polypyridyl complexes are ideally suited to this task. The present experiments reveal different binding interactions of **1** with G4 structures formed in solution by the human telomer sequence and show that these interactions depend on both the enantiomer and the particular folding topology of the polymorph structures formed in the presence of Na or K cations. The visible absorption G4 binding studies show significant changes in the 11,12-dCN-dppz ^1^MLCT transition at 440 nm, which are used to establish the different affinity of the complex in the different systems (Table 1). These studies report on the extent to which the environment of the dppz ligand changes in the presence of the G4 structure and indicate a greater binding affinity between Λ-**1** and the quadruplex structures Λ-**1**/**htel(K)** > Λ-**1**/**htel(Na)** > Δ-**1**/**htel(K)** > Δ-**1**/**htel(Na)**. This trend is in agreement with the greater affinity of the lambda enantiomer previously observed for the related Λ-[Ru(bpy)_2_dppz]^2+^ and mononitrile Λ-[Ru(phen)_2_dppz-CN]^2+^ for the **htel(K)** structure.^90,75^ The extracted binding constants (Table 1) indicate that the lambda enantiomer has a significantly greater preference for the biologically relevant **htel(K)** structure over the **htel(Na)** structure, with a 1 order of magnitude difference in the calculated *K*_b_ values; (1.1 ± 0.6) × 10^7^ M^–1^ versus 2.2(±0.3) × 10^6^ M^–1^. This is attributed to a complementarity of the enantiomer with the loop arrangement in the **htel(K)** compared to the **htel(Na)** structure, which allows for greater stacking interactions of the dppz ligand with the terminal guanine tetrads.^58^ In contrast, the delta enantiomer exhibits lower and comparable affinities for the two structures ca. (2–4) × 10^5^ M^–1^, which agrees with the previous observations for the enantiomer binding of related complexes to **htel(K)**. The comparable affinity exhibited for **htel(Na)** is attributed to the greater flexibility of this structure and the presence of loop interactions, which may hamper stacking of dppz with the guanine tetrads. Notably, the estimated binding free energy (Δ*G*_binding_) obtained by averaging over the three 350 ns simulations (Tables S4, S6, S9 and S10) also predicts a greater affinity of Λ-**1** for both structures: Λ-**1**/**htel(Na)** −78.23 kcal mol^–1^, Λ-**1**/**htel(K)** −75.52 kcal mol^–1^, Δ-**1**/**htel(K)** −73.79 kcal mol^–1^, Δ-**1**/**htel(Na)** −60.13 kcal mol^–1^. Interestingly, in contrast to the solution studies of Λ-**1**, the calculated binding energies suggest a stronger affinity for **htel(Na)** over **htel(K)**. This may be due to inclusion of highly favorable loop interactions available in one specific arrangement in the binding pocket that is not prevalent in solution. Overall, these results indicate the potential of exploiting enantiomers of octahedral transition metal complexes to detect quadruplex structures with different loop topologies.

The time-resolved infrared experiments show that when either Λ-**1** or Δ-**1** is bound to the hybrid **htel(K)**, the formation of the excited state almost exclusively perturbs the guanine bases with a notable absence of strong bleaches associated with adenine and thymine bases (Figure 4). In the case of Λ-**1**, the spectrum is similar to one previously observed for the racemic mixture of the parent [Ru(phen)_2_dppz]^2+^ bound to **htel(K)**.^58^ These observations can be rationalized in terms of the expected response to the MLCT nature of the excited state, as the TRIR “site effect” is expected to report on bases that are in close proximity to the Ru(II) center or the dppz ligand where the CT occurs (Figure 1). The strong interaction of the 11,12-dCN-dppz ligand with the guanine bases are in agreement with the favored binding positions identified by the MD binding analysis where the 11,12-dCN-dppz is observed to stack with the lower G-quartet while the ancillary ligands are found to form some contacts with the loops. The structure obtained for Λ-**1** bound to **P7** shows intercalation from below with favorable contacts made with the guanine bases (Figure 7a). Structural studies on the binding of the related mononitrile complex [Ru(TAP)_2_(11-CN-dppz)]^2+^ with quadruplex DNA revealed polar contacts between the nitrile substituent and a 2-NH_2_ on guanine, which act to enhance the binding interaction.^75^ Interestingly, the approach of Δ-**1**, which intercalates from the adjacent side at **P6**, positions the nitrile group in close contact with a guanine in the bottom G-quartet. The formation of a polar contact here may explain the appearance of 1690 cm^–1^, which is attributed to a shift in a guanine carbonyl vibration to higher wavenumber due to a polar interaction between a guanine base and the nitrile group (Figure 7b and Figure 4b). Previously we have reported the appearance of a transient band at higher wavenumbers associated with the formation of the guanine radical cation. In these studies with double stranded DNA^53^ and related transient visible absorption studies by the Thomas group on quadruplex DNA,^91^ a grow-in of the diagnostic band was observed over hundreds of picoseconds. The instantaneous perturbation observed here is more consistent with the site effect previously observed for the TRIR study of the [Ru(phen)_2_dppz].Cl_2_ light switch complex, which is unable to photo-oxidize guanine.^58^ Furthermore, if rapid photo-oxidation was occurring, this would also be expected to occur for the Λ-**1** binding site.

In addition to the key interactions in the G4 structure, the nitrile probe can be exploited to provide additional information on the binding site. The sensitivity of the ground state nitrile vibration to the hydrogen bonding environment has been previously highlighted in the work of Boxer^63,67,92^ and the work of Bagchi,^65^ which have exploited the location of the nitrile band to report on biologically relevant systems. In our study, a significant photoenhancement of the nitrile stretching vibration is observed for the MCLT excited state of **1** formed upon 400 nm excitation (Figure 3). A similar enhancement has been observed for aromatic nitriles^80^ and CN substituted bipyridyl complexes of ruthenium(II).^81,82^ However, this is the first time that such an effect is reported for an intercalating probe. This enhancement serves to amplify the environmental response of the nitrile vibration, which is found to display a linear shift to the DMSO/water environment and builds on observations of the solution response of nitriles in the ground state.^65^

Applying this to the hybrid **htel(K)** binding, the shift in the nitrile position provides a highly localized reporter on its environment and access of the nitrile solution HB for Λ-**1** compared to Δ-**1** (Table 2). Interestingly, the lifetime is found to be enhanced for both enantiomers by a similar extent. This reflects the access of the phenazine nitrogen to the protic solvent, which from the MD simulations is expected to be similar. This is remarkable as we are now combining the nitrile shift and the observed kinetics to report separately on the environment of the central and distal regions of the 11,12-dCN-dppz ligand. The TRIR spectra obtained for Λ-**1** or Δ-**1** bound to the antiparallel **htel(Na)** show striking differences in the nucleobase bleaches, which immediately signal a different binding environment^58^ (see Figure 5a–b). The TRIR spectrum of Λ-**1**/**htel(Na)** is dominated by guanine interactions indicated by the strong guanine bleach at 1675 cm^–1^. In contrast, the TRIR of Δ-**1**/ **htel(Na)** indicates a greater relative intensity of the thymine (ca. 1640 cm^–1^, 1662 cm^–1^, 1705 cm^–1^)^84^ and adenine (ca. 1620 cm^–1^) bleach bands. The MD simulations indicate that these differences arise due to differences in the penetration of the complex into the G4 structure, which is restricted by loop interactions (see Figure 9). Binding of Λ-**1** to **P4** shows favorable interactions with the G-quartets and some interaction of the 11,12-dCN-dppz with thymine and adenine nucleobases (Figure 9a). In contrast, in the case of Δ-**1** in the **P1** there is weaker interaction with the guanine bases and a relatively greater contribution of the loop interactions. The difference in the two binding environments is reflected in the marked change in the position of the frequency of the nitrile transient, which is correlated to the access of the nitriles to the aqueous HB environment (Figure 5c–d). Notably, binding to the antiparallel **htel(Na)** results in greater differences in the lifetimes determined for the decay of the excited state (Figure 5e–f), which indicates a different environment for the central phenazine part of the 11,12-dCN-dppz ligand and is also supported by the MD simulations (Figure 9). The excited state dynamics of [Ru(phen)_2_dppz)]^2+^ type complexes are known to be very sensitive to the nature of DNA binding.^93^ Across the four systems, biexponential kinetics are observed, which indicate that in solution the complex experiences more than one environment of similar binding affinity. Interestingly, the interactions of the enantiomers also increased the stabilizations of the DNA structures, shown through a reduced variation in the RMSD over the simulation time (Figure S15 and Figure S17).

## Conclusion

In conclusion, this study reveals the origin of differences in binding interactions between the two enantiomers of the DNA intercalating complex [Ru(phen)_2_(11,12-dCN-dppz)].Cl_2_, to two different G-quadruplex conformations. Critically, the amplified signal of the nitrile vibration of the excited state is shown to be sensitive to its hydrogen bonding environment, which also impacts the relaxation dynamics. This effect is further enhanced by the location of the nitrile vibration in “transparent window” between 1800 and 2500 cm^–1^, which is well separated from the congested spectral regions of biological macromolecules such as proteins and DNA.^94^ This sensitivity is combined with (i) kinetic analysis and (ii) the identity of the bases in the binding site determined by the perturbation related “site effect”, to provide a detailed picture of the G4 binding environment. Furthermore, these solution-based observations are complemented by detailed MD simulations. Very recently the photoenhancement of the nitrile vibration intensity of aromatic nitriles was reported, and it was suggested that these could in future be applied as sensitive probes.^80^ The work reported here is a beautiful demonstration of this and the first time that the photoenhancement of the nitrile vibration in the excited state has been exploited to act as an amplified IR probe of a biological environment.

G4 structures have been shown to accommodate the presence of oxidized bases,^95^ and it is speculated that guanine oxidation in quadruplexes may be linked to epigenetic response.^96,97^ This raises the possibility to use structurally related G4 targeting complexes containing IR probes to report directly on the impact of guanine photo-oxidation on the G4 structure. Furthermore, IR probes are finding increasing application in cellular studies and were recently used to image metabolic processes.^98^ Related to this, we have recently reported the use of TRIR to monitor the excited states of probes in cells.^99^ Now this current study points to the potential of exploiting excited state amplification of the IR probe signal to monitor cellular processes, including processes related to the development of inorganic based diagnostics and therapeutics.

## Experimental Section

[Ru(phen)_2_(11,12-dCN-dppz)](PF_6_)_2_ (11,12-dCN-dppz = 11,12-dicyanodipyrido[3,2-a:2′,3′c]phenazine) (**1**) was prepared using previously reported methods.^100^ The enantiomers were resolved by passing through a C25-sephadex column eluted with a (−)-O,O′-dibenzoyl-l-tartrate mobile phase. The purity of all ligands was confirmed by ^1^H NMR spectroscopic analysis. All other chemicals employed were of reagent-grade quality and were used without further purification. The oligonucleotide were synthesized, desalted, and purified (by gel filtration) by Eurogentec (Liege, Belgium). Oligonucleotide and DNA concentrations were determined spectrophotometrically.

### DNA Titrations

The concentration of DNA was determined using the molar absorbance at 260 nm for G4 (244300 M^–1^ cm^–1^ /single-strand). UV/vis and emission titrations were carried out at **1** at 298 K by monitoring changes in the absorption and emission spectra of the complexes upon successive additions of aliquots of DNA, in sodium phosphate buffer (20 mM, pH 7.0). The results are quoted using the concentration of DNA expressed as a concentration of G4 tetrad [DNA] to Ru ratio ([DNA]:Ru ratio).

### Instrumental Methods

^1^H NMR spectra were obtained on a Varian VnmrS 400 MHz spectrometer. All electrospray ionization mass spectrometry (ESI-MS) studies were performed by using an Agilent 6546 Q-TOF series LC/MS system. UV/vis absorption spectra were recorded on a Varian Cary 200 spectrophotometer and a Varian Cary 50 spectrophotometer. Steady-state luminescence spectra were recorded on a Varian Cary Eclipse. Circular dichroism measurements were recorded on a JASCO J-810 spectropolarimeter. Time Resolved spectroscopy measurements were conducted on an ULTRA and Time-resolved Probe Spectroscopy (TRMPS) apparatus at the Central Laser Facility (STFC Rutherford Appleton Laboratory, Harwell, UK), which is described in detail elsewhere.^101^ During experiments the samples were raster scanned in the *x* and *y* directions to minimize photodamage and re-excitation effects. The samples were excited at 400 nm for the ruthenium complexes. All experiments were carried out at 298 K, and samples were checked before and after the experiment by UV–visible spectroscopy (PerkinElmer lambda 950 spectrophotometer) and FTIR in a Nicolet Avatar spectrometer.

The time resolution for these experiments was 150 fs in the picosecond time domain and 1 ns in the nanosecond time domain. For the acquisition of the spectra, the polarization of the pump pulses at the sample was at the magic angle relative to the probe and was attenuated to 1 μJ. For ULTRA picosecond and nanosecond measurements the pump beam was mechanically chopped to 5 kHz and focused to an ∼100 μm diameter spot size and overlapped with the probe beam (∼50 μm diameter spot size) in the sample cell. This produced two probe pulses for every one pump pulse: one probe pulse of the sample after laser excitation and another probe pulse of the sample with no laser excitation, which were used to generate the difference spectra at each time delay. Samples were loaded into a demountable Harrick cell (Harrick Scientific Products Inc., New York) assembled with 25 mm diameter, 2 mm thick CaF_2_ plates (Crystran, Ltd., UK), separated by a 50 μm Teflon spacer in D_2_O and recorded in a N_2_ flushed transmission accessory. Each spectrum is an average of 32 scans. **TRIR calibration:** All spectra were processed using the in-house Ultraview software provided by the central laser facility (CLF). Samples were calibrated from pixels into wavenumbers by using the Ultracal software. The nitrile band region (2150–2300 cm^–1^) was calibrated by fitting the absorption bands of acetonitrile, and the remaining metal complex and DNA region (1250–1860 cm^–1^) was calibrated by fitting to the absorption bands of polystyrene.

### Computational Modeling

#### Classical Molecular Dynamic (MD) Simulations

The enantiomers of the complex of **1** were constructed based on the crystal structure of the related complex [Ru(TAP)_2_(dppzCN)]^2+^ reported by Cardin et al.^75^ in the crystal structure PDB: 5LS8, which was used as a template. After construction, the ground state geometries of both complexes were optimized at the Density Functional Theory (DFT) level of theory using the PBE0 functional,^102^ def2-svp basis set^103^ and using a Douglas–Kroll–Hess scalar relativistic Hamiltonian as implemented in the Gaussian16 suite, v.A03.^104^ The octahedral ruthenium coordination sphere of Λ-**1** and Δ-**1** complexes was described with force field (FF) parameters at the general amber force field (GAFF), with *ad hoc* parametrized charges obtained from restrained electrostatic potential calculation on the optimized ground state geometries. MD simulations of the human telomere sequence d[AG_3_(TTAG_3_)_3_] were performed using the hybrid conformation (stabilized by K^+^ ions) (PDB: 2HY9)^105^ (with two additional adenines located at both termini removed) and the antiparallel conformation (stabilized by Na^+^ ions) (PDB: 143D).^77^ The G4 DNA was described by OL15 FF.^106^ Two additional K^+^ cations were manually placed in between the G-quartets. This system was then neutralized by the addition of counterions and immersed in a periodic water box filled with TIP3P water molecules using *tleap*.^107^ This process was repeated for the antiparallel structure using Na^+^ ions. Periodic boundary conditions were used for all systems, and long-range electrostatic interactions were solved via the particle mesh Ewald method with a grid spacing of 1 Å and a cut off of 10 Å. All the hydrogen atoms were restrained using the SHAKE algorithm.^108^

Initially, due to the absence of the K^+^ and Na^+^ ions on the relaxed hybrid and antiparallel structures solved by NMR, a 100 ns MD was run to equilibrate and relax both unbound structures. After that, complexes Λ-**1** and Δ-**1** were manually positioned into 7 feasible binding pockets on the relaxed hybrid **htel(K)** structure and into 8 possible binding pockets on the relaxed antiparallel **htel(Na)** structure. In all cases, all the MD simulations underwent the following steps: (**1**) The minimization consisted of three stages; a) first, the hydrogens; b) second, the solvent molecules (waters and counterions); and c) third, the entire system was sequentially minimized in 20,000 steps. For the initial 10,000 steps, a steepest descendent algorithm was used; for the final 10,000 steps, a conjugate gradient algorithm was used. (**2**) The solvated geometries were heated from 100 K (at which temperature initial velocities were assigned) to 300 K in 20 ps using the Langevin thermostat, with an integration step of 2 fs (collision frequency of 1 ps^–1^). The positions of all heavy atoms in the system were restrained with a strong harmonic constant (40 kcal^–1^ mol^–1^ Å^–2^). This step included a random seeding approach, where the Langevin dynamics and the initial velocity of the dynamics were dependent on a random number. (**3**) The restraints were progressively loosened and then removed within six steps of 20 ps each, where the system was switched from an NVT (40 to 10 kcal^–1^ mol^–1^ Å^–2^ in 80 ps, constant volume) to an NPT (10 to 0 kcal^–1^ mol^–1^ Å^–2^ in 40 ps, constant pressure) ensemble. (**4**) Each of the probe–DNA systems was then simulated at 300 K for 100 ns and constant pressure (1 atm), using the pmemd.cuda engine of single-precision - fixed precision (SPFP) on two GeForce Nvidia GTX 1080Ti GPUs. An integration time step of 2 fs was defined, and coordinates were compressed and saved every 10 ps. The results of these simulations provided a realizable binding mode for complexes Λ-**1** and Δ-**1** to each of the G4 structures. The selection was made by visual inspection and binding energy analysis. The interactions of each MD simulation were then analyzed using the MM-ISMSA approach.^86^ This approach consisted of calculating the gain in Gibbs free energy after binding by a thermodynamic cycle. This cycle consisted of calculating, at the FF level, first the binding energy in vacuum, considering the gain in energy when the probe and the G4 DNA form the complexes, then a solvation term is calculated for probe, DNA, and complex by considering a polar and a nonpolar contribution. The first one considered the gain in energy in changing the phase from gas to solution. The second considered the gain in energy of the attractive probe–DNA interaction with respect to the loss of energy due to the formation of the binding pocket. In MM-ISMA the solvation term was calculated with an implicit solvent model, notably speeding up the calculation. The energy calculation was performed for a statistically relevant number of conformers and the final Δ*G*_binding_ averaged over those. The binding mode with the most energetic interactions was then run for three further independent 350 ns extended MD simulations, initializing each of them with different velocities randomly assigned, to further analyze the structural stability of this binding position. Results were then visualized using PyMOL Version 2.5.4 and Gnuplot 5.5 software. The stability of the structural parameters of the G4 upon binding were further investigated following the approach described by Tsetkov et al.^88^ and running the script provided by the authors, adapting the residue numbers to our system. The script was run for the 350 ns long dynamics, producing the most stable binding mode. A snapshot was extracted every nanosecond to give 350 snapshots for the analysis for each complex. Each of the snapshots is composed of the G4 and the probe and the first solvation shell.

## Supporting Information Available

Additional information on the synthesis and characterization of the system including spectroscopic and computational data is available. (PDF)
