# Supplementary material for: Good Vibrations Report on the DNA Quadruplex Binding of an Excited State Amplified Ruthenium Polypyridyl IR Probe
Source: J Am Chem Soc. 2023 Sep 22;145(39):21344–60. doi: 10.1021/jacs.3c06099 (PMC10557146; doi:10.1021/jacs.3c06099)
Supplement: Supplementary file 1 — ja3c06099_si_001.pdf [file ja3c06099_si_001.pdf]

Good Vibrations Report on the DNA Quadruplex Binding of an Excited State Amplified Ruthenium Polypyridyl IR Probe

Mark Stitch,[a] Davide Avagliano,[b,c] Daniel Graczyk,[a] Ian P. Clark,[d] Leticia González,[b,e]* Michael Towrie,[d] and Susan J Quinn[a]*

[a]*School of Chemistry, University College Dublin, D04 V1W8, Ireland.*

[b]*Institute of Theoretical Chemistry, Faculty of Chemistry, University of Vienna, Währingerstr. 19, 1090 Vienna Austria*

[c]*Department of Chemistry, Chemical Physics Theory Group, 80 St. George St., University of Toronto, Ontario M5S 3H6, Canada*

[d]*Central Laser Facility, STFC Rutherford Appleton Laboratory, Harwell Science and Innovation Campus, Didcot, Oxfordshire OX11 0QX, U.K.*

[e]*Vienna Research Platform on Accelerating Photoreaction Discovery, University of Vienna, Währingerstr. 19, 1090 Vienna Austria*

Synthesis of [Ru(phen)$_2$(11,12-dCN-dppz)]Cl$_2$[1]

11,12-Dicyanodipyrido-[3,2-a:2',3'-c]phenazine (11,12-dCN-dppz) (115.7 mg, 0.35 mmol) and [Ru(Phen)$_2$Cl$_2$] (185 mg, 0.355 mmol) were added to a solution EtOH/H_2O (50 ml; 1:1). The mixture was refluxed overnight and the solution turned black. The solvent was then removed using a rotary evaporator and the crude product was purified using column chromatography on a silica gel with MeCN/H_2O/$NaNO_3$ (40:4:1). The fractions containing the product were combined and the solvent was removed. The precipitate was then dissolved in 30 ml MeCN and filtered to remove the insoluble $NaNO_3$. The solvent was again removed, and the product was dissolved in 30 ml deionized water and NH_4PF_6 was added to collect the suspended [Ru(phen)$_2$(11,12-dicyanodipyrido-[3,2-a:2',3'-c]phenazine)](PF_6)$_2$ as the PF_6^- salt. The precipitate was washed with deionized water and diethyl ether and dried under vacuum. To obtain the purified [Ru(phen)$_2$(11,12-dCN-dppz)](PF_6)$_2$ (200 mg, 71% yield). **^{1}H NMR (400 MHz, Acetonitrile-*d*$_3$)** δ 9.58 (dd, *J* = 8.2, 1.3 Hz, 2H), 9.07 (s, 2H), 8.63 (ddd, *J* = 8.2, 6.7, 1.3 Hz, 4H), 8.28 (s, 4H), 8.24 (dd, *J* = 5.3, 1.3 Hz, 2H), 8.18 (dd, *J* = 5.4, 1.4 Hz, 2H), 8.02 (dd, *J* = 5.3, 1.3 Hz, 2H), 7.81 (dd, *J* = 8.3, 5.4 Hz, 2H), 7.66 (ddd, *J* = 16.0, 8.3, 5.3 Hz, 4H).

[Ru(phen)$_2$(11,12-dCN-dppz)](PF_6)$_2$ (200 mg) was converted to the water-soluble chloride salt by dissolving the complex in 40 ml of amberlite resin Cl ion exchange beads, which had previously been soaked in methanol overnight. The suspension was allowed to sit overnight with constant swirling every half an hour. Following this, the ion exchange beads were filtered off and the solvent was removed by rotary evaporator and the dark brown solid was collected (93.5 mg). Ion exchange was confirmed using ^{31}P NMR.

^{1}H NMR (400 MHz, Deuterium Oxide) δ 9.48 (d, *J* = 8.2 Hz, 2H), 8.93 (s, 2H), 8.48 (t, *J* = 7.9 Hz, 4H), 8.18 – 8.09 (m, 8H), 7.94 (d, *J* = 5.5 Hz, 2H), 7.65 (dd, *J* = 8.3, 5.4 Hz, 2H), 7.50 (ddd, *J* = 24.6, 8.3, 5.3 Hz, 4H). **^{13}C NMR (101 MHz, cd$_3$cn)** δ 155.53, 153.35, 152.98, 151.84, 147.83, 147.76, 143.45, 142.49, 137.97, 137.08, 137.02, 134.00, 131.10, 131.08, 129.89, 128.09, 127.66, 125.96, 125.90, 117.31, 115.36, 115.24. ATR CN band stretch – 2230 cm^{-1}

Chiral Resolution of [Ru(phen)$_2$(11,12-dCN-dppz)]$^{2+}$

The resolution of the enantiomers of [Ru(phen)$_2$(11,12-dCN-dppz)] was achieved by a modification to the literature methods.[2, 3] A solution of the racemic complex as its chloride salt was dissolved in a minimum amount of water and loaded onto the 1 m Sephadex column. The complex was recycled a total of 3 times (in darkness) using a peristaltic pump to allow complete separation using a 0.1 M sodium tartrate mobile phase (reduced to 0.01 M for final cycle). During recycling, the speed of the pump was reduced to 0.1 mL min^{-1} to minimise mixing of the enantiomers in the capillary tubes. After complete recycling of the complex, the flow rate was increased to its original speed of 1 ml min^{-1}. This process was continued for a number of cycles until the Λ- and Δ-enantiomers were observed as two separate bands on the column (Shown in figure S12).

The tartrate salts obtained were converted to the chloride salts by gently shaking the fractions with amberlite ion exchange resin (chloride form) beads and the removal of tartrate was monitored by UV/vis. The PF_6^- salt of the enantiomer was then collected by the addition of concentrated solution of NH_3PF_6 and the collected salt was then washed with an excess of water (to remove any excess NaCl from the exchange process). The chloride form was then regenerated by swirling a solution of the complex in MeOH in the presence of Amberlite for 1 h. The beads were then removed, and the solvent removed under reduced pressure and the resulting red solid was dried under high vacuum. Identification of each enantiomer was achieved by CD spectroscopy and the Δε values of each enantiomer were compared to ensure adequate resolution.

Binding calculation: Using the method described by Bard *et al.*[4] in the equation shown below (**1**). The binding constant of the enantiomers binding to DNA structures was determined by fitting equation (**1)** to a non-linear plot of $(\varepsilon_a - \varepsilon_f)/(\varepsilon_b - \varepsilon_f)$ vs [DNA] (in concentration of G-tetrads), using Origin 8.5 software.

$$\frac{(\varepsilon_a-\varepsilon_f)}{(\varepsilon_b-\varepsilon_f)} = (b - (b^2 - 2k^2 C_t[DNA]/s)^{\frac{1}{2}})/2KC_t \quad (\mathbf{1})$$

$$b = 1 + KC_t + K[DNA]/2s \quad (\mathbf{2})$$

Where; [DNA] is the concentration of DNA in G-tetrads; ε_a, ε_f and ε_b are the apparent extinction coefficient (A_{abs}/[M]), the extinction coefficient of free metal (M) complex and the extinction coefficient of the metal (M) complex in the fully bound form, respectively; K is the equilibrium binding constant in M^{-1}, C_t is the total metal complex concentration, and s is the binding site size (per G-tetrad).

Figures SI

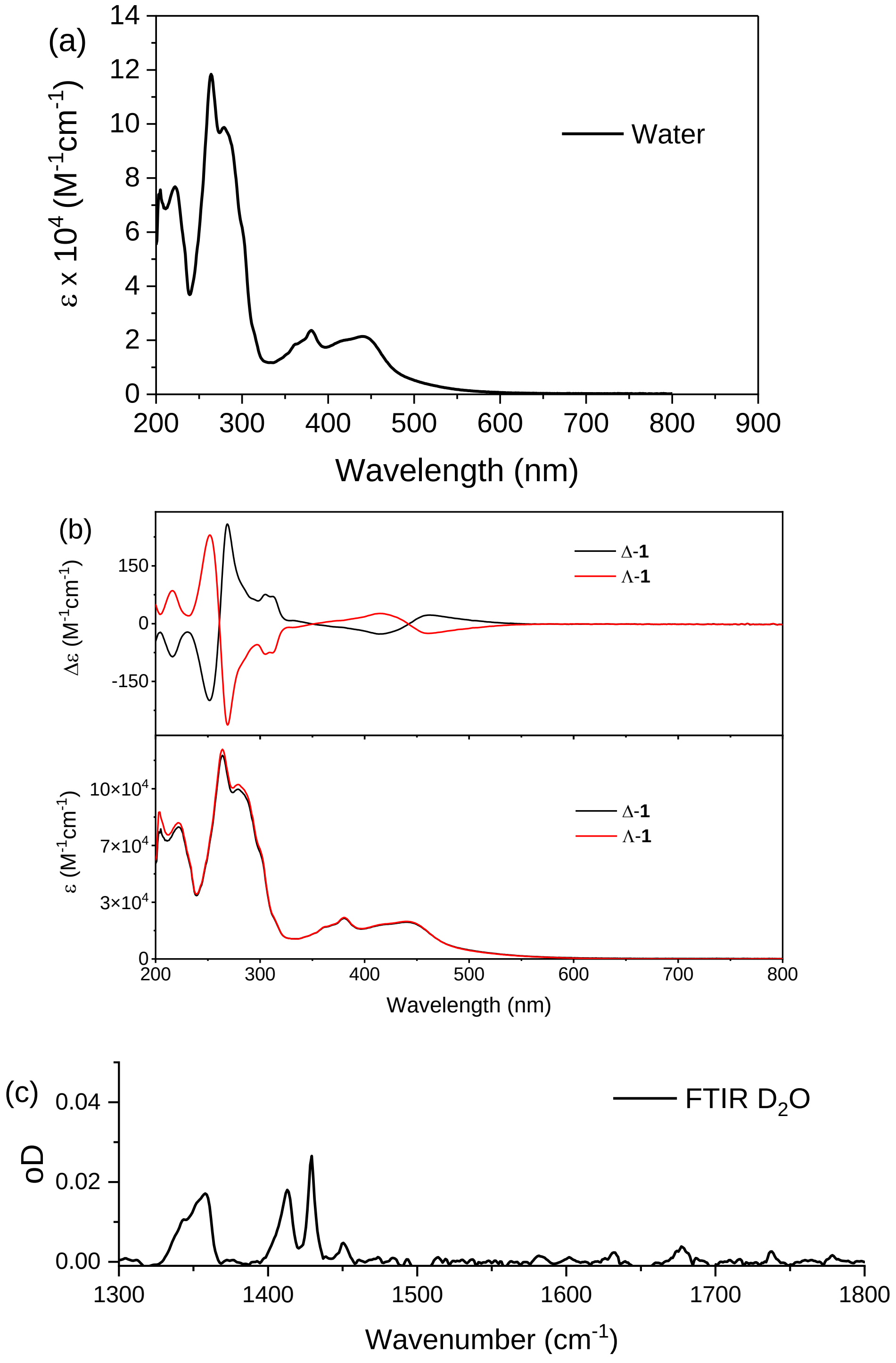

Figure S1 (a) UV-Vis spectrum of the Δ-**1** and Λ-**1** enantiomers in water (No detectable emission in MeCN) (b) CD spectrum of the enantiomers. (c) FTIR spectrum of *rac*-**1** (1 mM) recorded in D_2O.

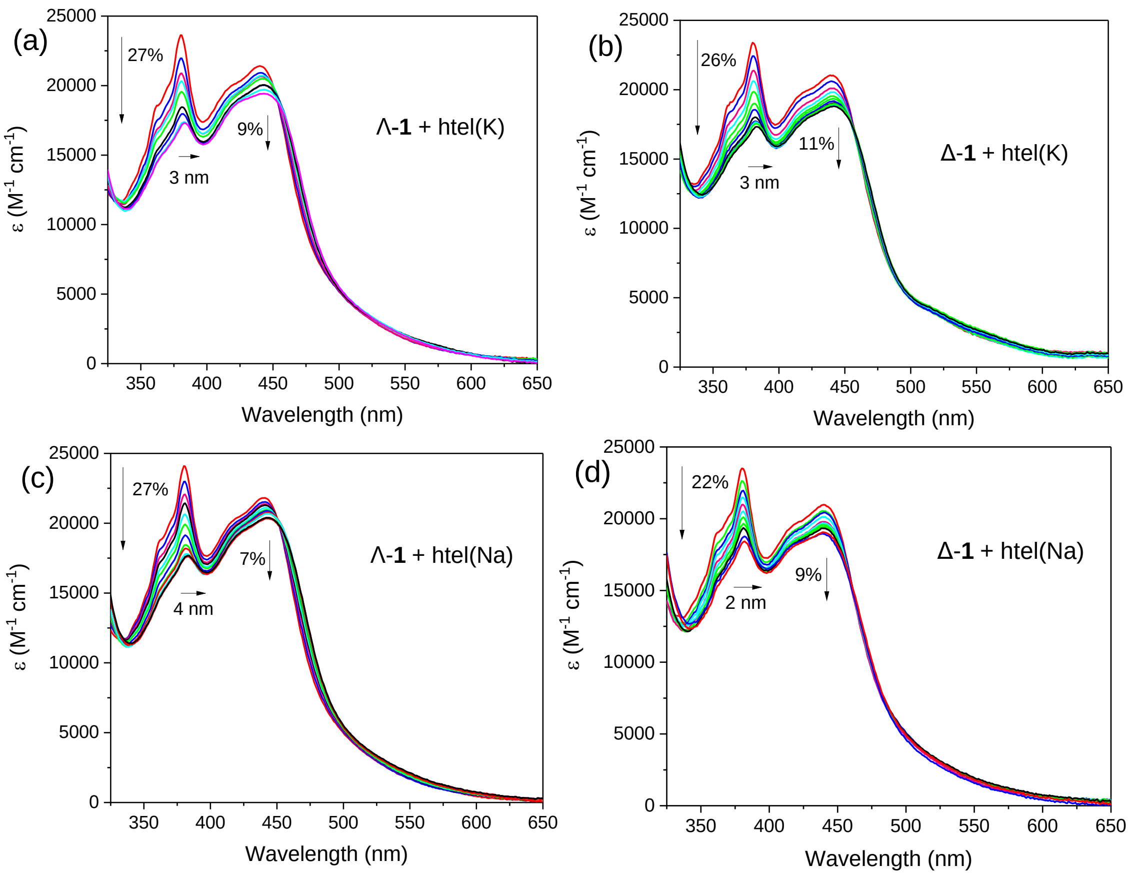

Figure S2: Absorbance spectra of (a)/(c) Λ–**1** (7.2 μM) and (b)/(d) Δ-**1** (5.6 μM) titrated against increasing concentrations of htel (0→37 μM) in 50 mM phosphate buffer and 100 mM KCl (**htel(K)**) or 100 mM NaCl (**htel(Na)**) at pH 7.

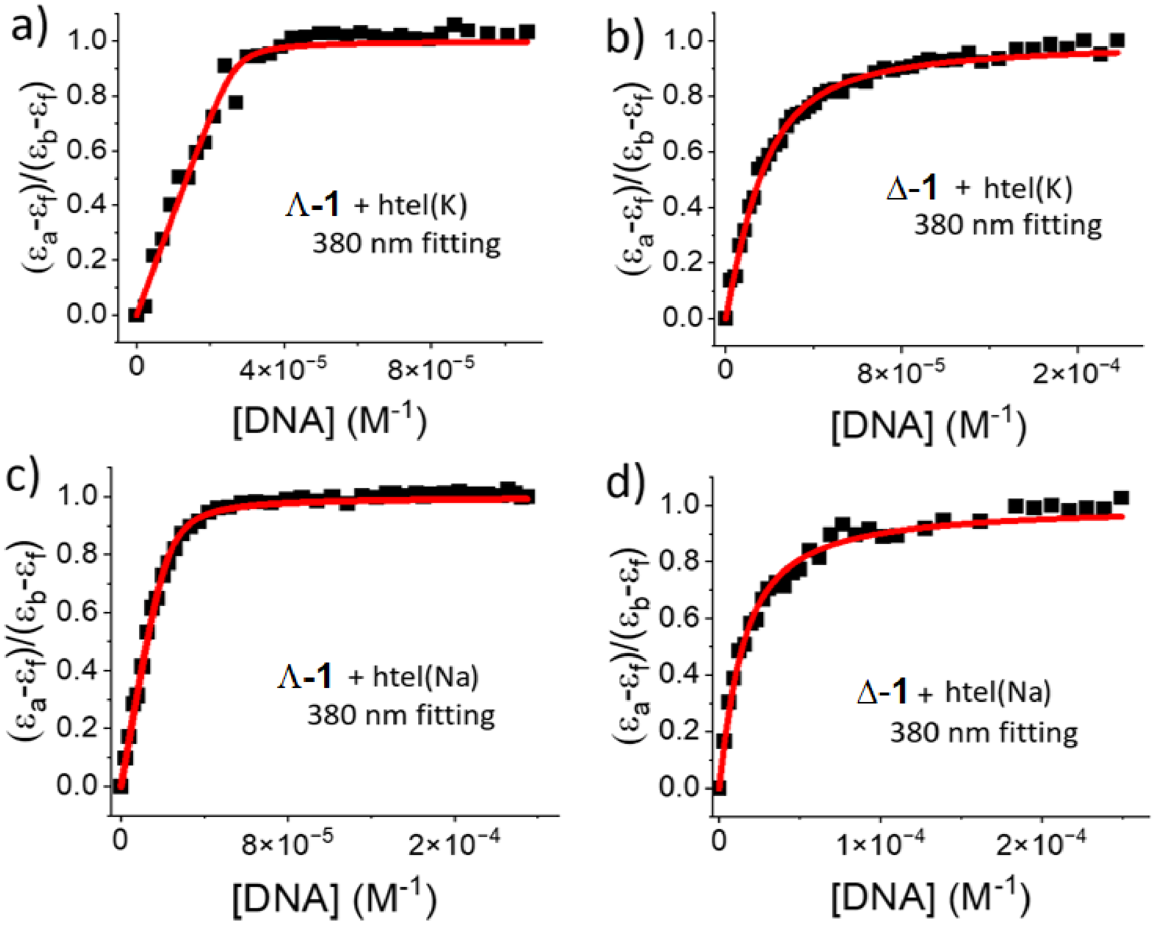

Figure S3. Determination of the binding constant for (a)/(c) Λ–**1** (7.2 μM) and (b)/(d) Δ-**1** (5.6 μM) in the presence of htel. Plots of $(\varepsilon_a-\varepsilon_f)/(\varepsilon_b-\varepsilon_f)$ fitting at 380 nm vs. [DNA] (per G-tetrad) and non-linear curve fitting of the data (**—**) using the method of Bard et al.[4] In 50 mM phosphate buffer and 100 mM KCl (**htel(K)**) or 100 mM NaCl (**htel(Na)**) at pH 7.0.

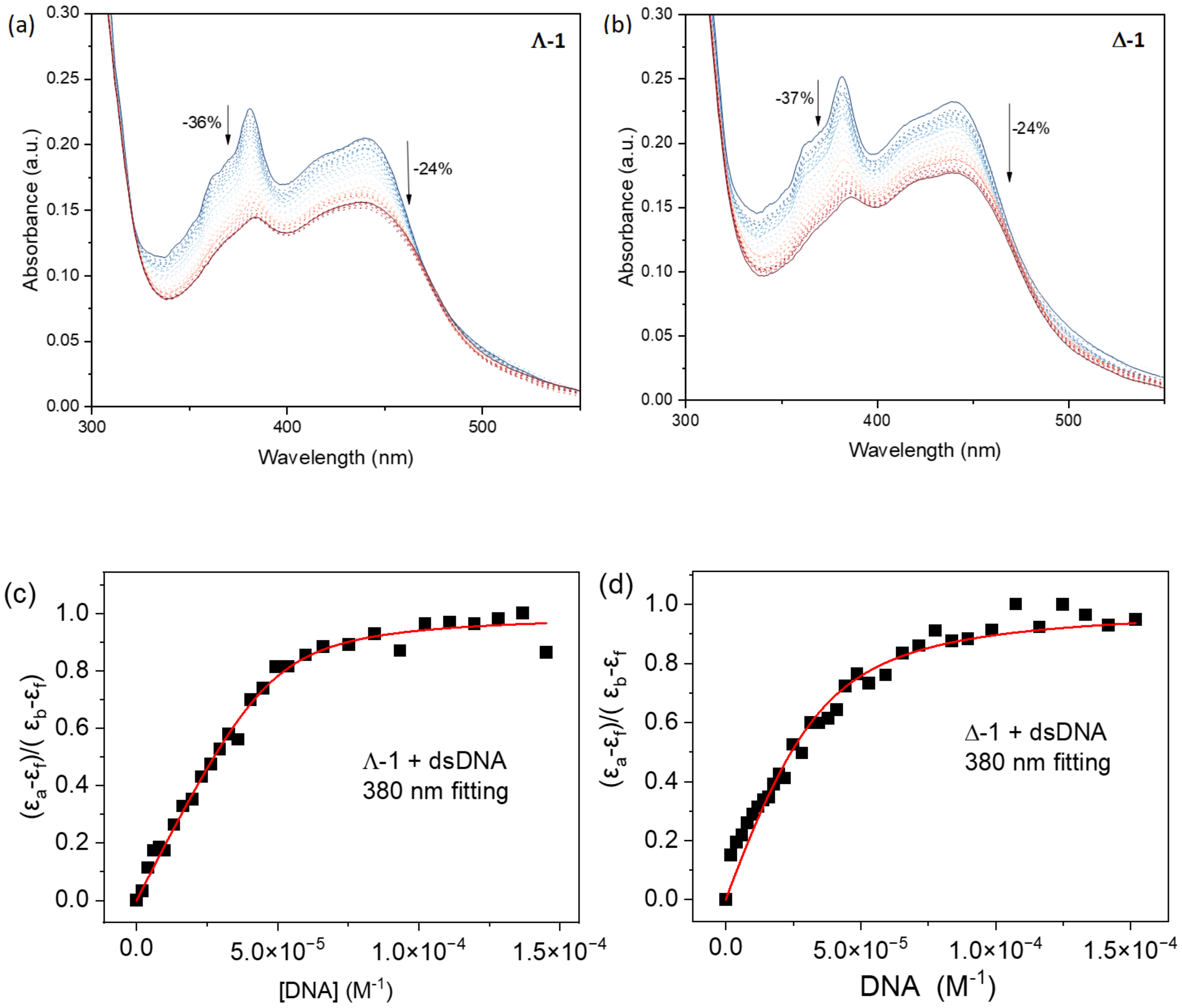

Figure S4. Absorbance spectra of (a) Λ–**1** (9.8 μM) and (b) Δ-**1** (11 μM) in the presence of salmon testes DNA double stranded DNA (dsDNA). Determination of binding constants (c) and (d) by plotting (ε_a-ε_f)/(ε_b-ε_f) fitting at 380 nm vs. [DNA] (per base pair) and non-linear curve fitting of the data (—) using the method of Bard et al.[4] In 50 mM phosphate buffer and 100 mM KCl at pH 7.0.

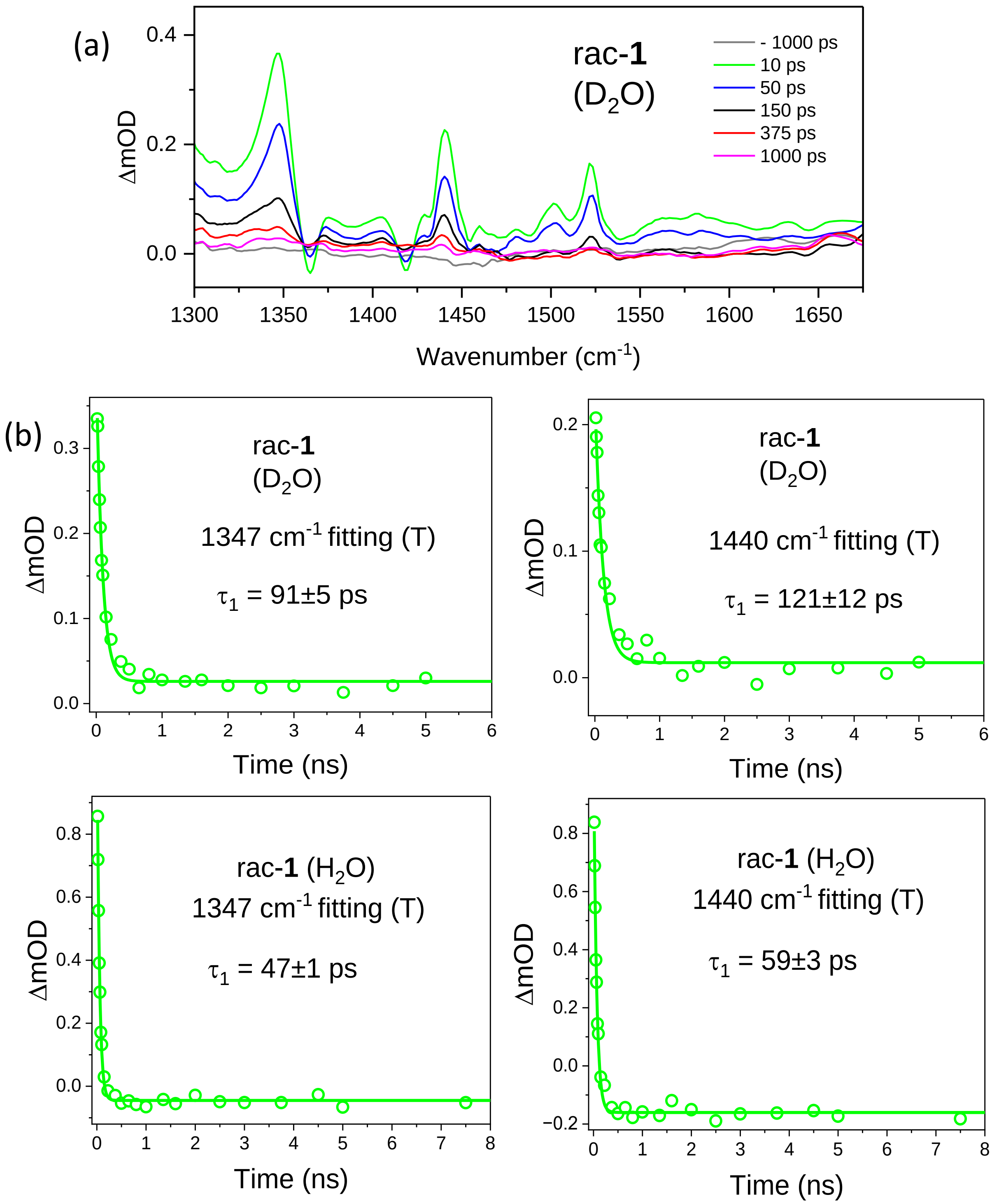

Figure S5: (a) TRIR difference spectra of 0.4 mM of rac-**1** in D_2O (λ_{ex}= 400 nm, 2 kHz,150 fs). (b) Kinetic analysis of the complex monitored at 1347cm^{-1} and 1440 cm^{-1} in both D_2O and H_2O.

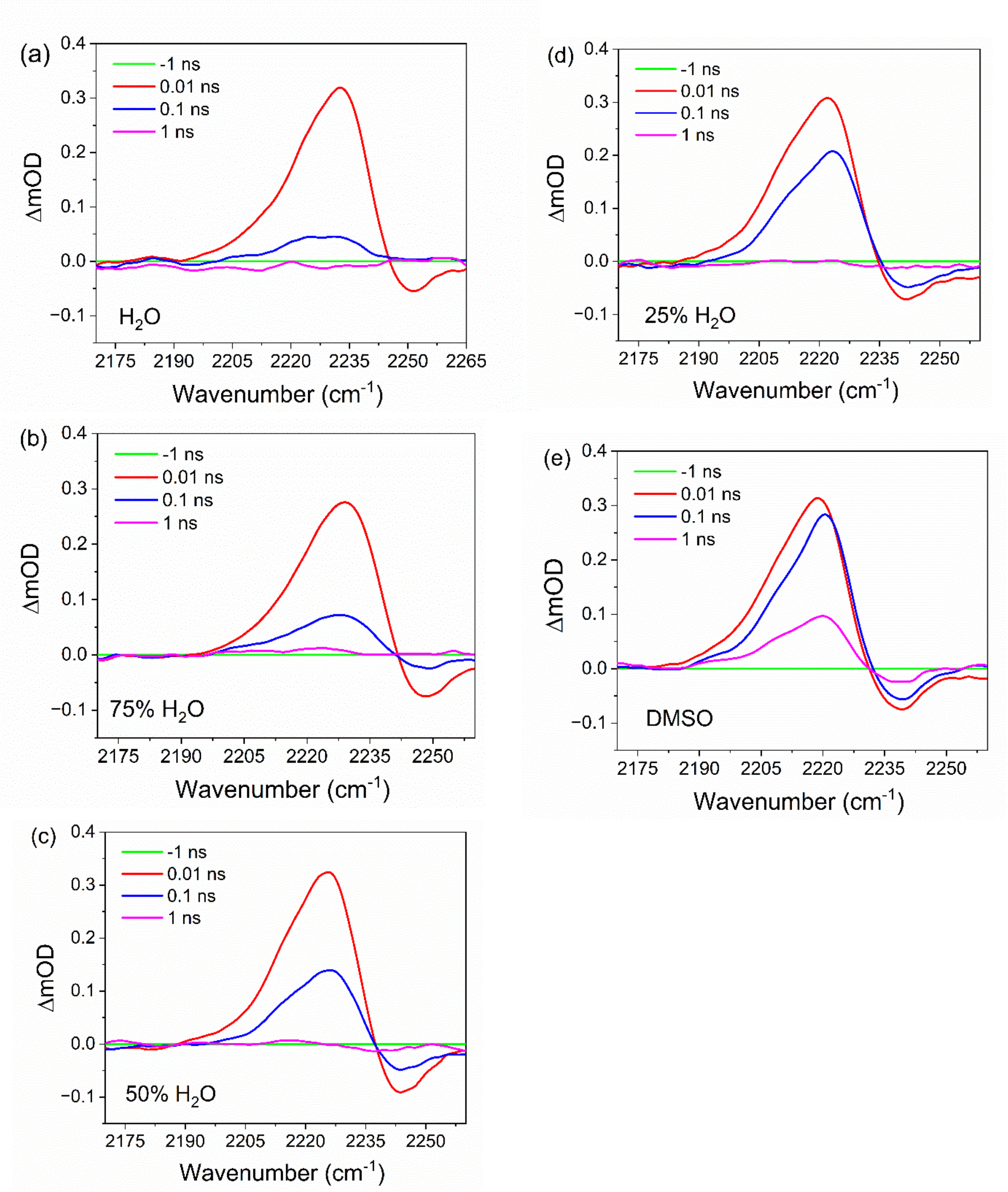

Figure S6: Solvent dependent nitrile response TRIR spectra of 1 mM rac-**1** recorded at 10 ps in different solution compositions of DMSO and water recorded after excitation, (λ_{exc} =400 nm). (a) H_2O, (b) 25% DMSO, (c) 50% DMSO, (d) 75% DMSO and (e) 100% DMSO.

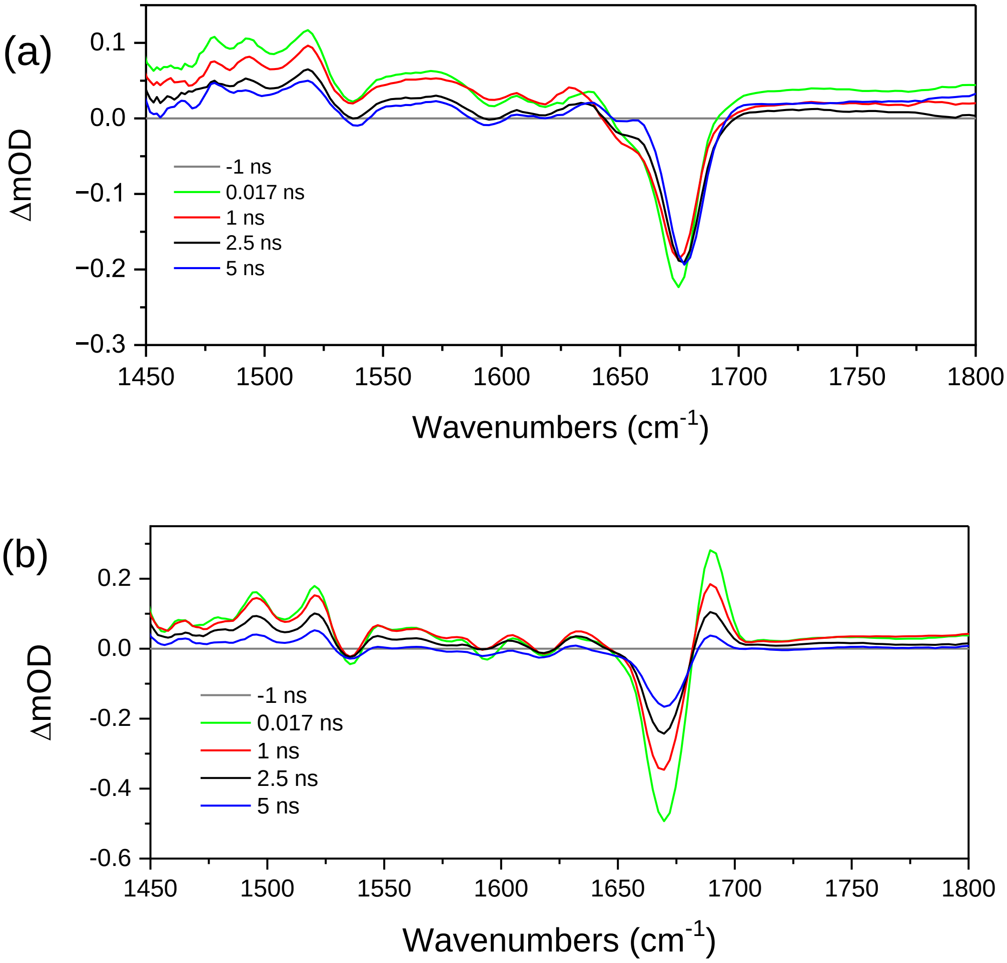

Figure S7: TRIR difference spectra of 0.4 mM of (a) Λ-**1** and (b) Δ-**1** in the presence of **htel(K)** in 50 mM K-phosphate, pH 7, in D_2O (λ_{ex}= 400 nm, 2 kHz,150 fs).

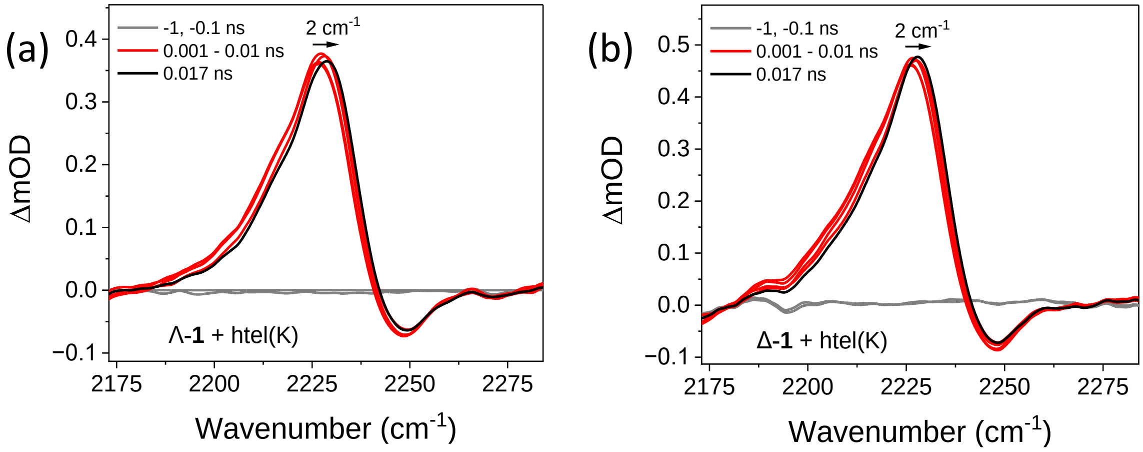

Figure S8: Vibrational cooling Nitrile band (a) Λ-**1** and (b) Δ-**1** when bound to **htel(K)**.

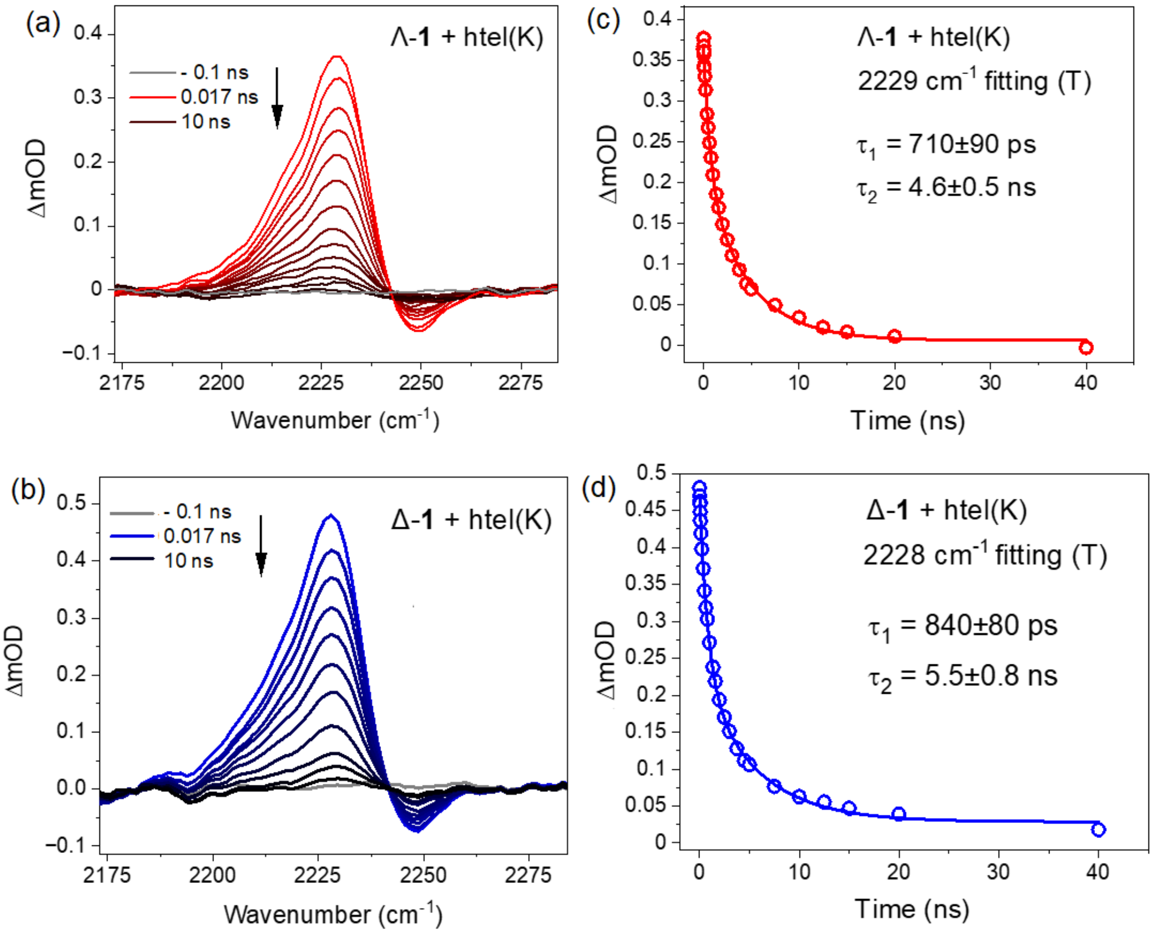

Figure S9: TRIR monitoring of the Nitrile band (a)/(c) Λ-**1** and (b)/(c) Δ-**1** when bound to **htel(K)**.

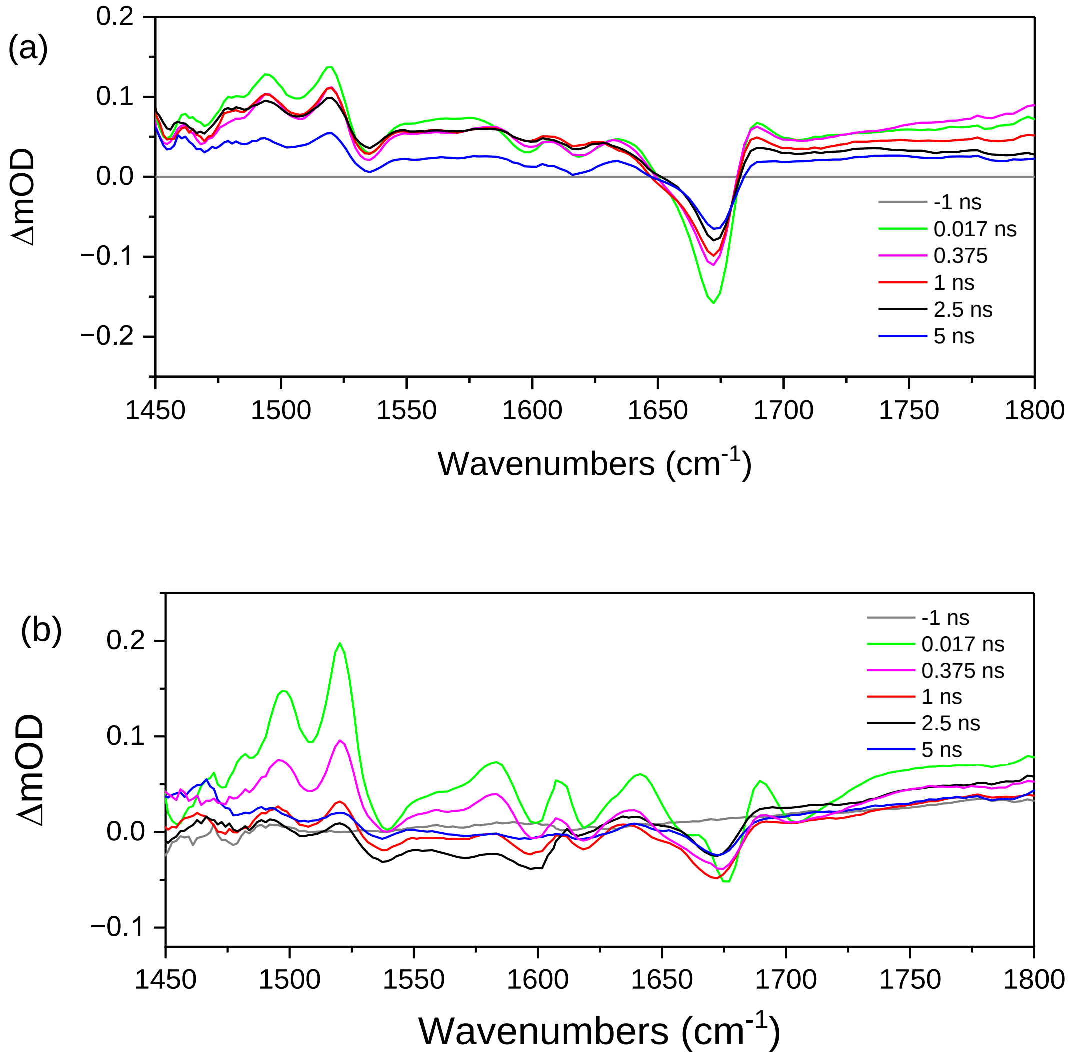

Figure S10: TRIR difference spectra of 0.4 mM of (a) Λ-**1** and (b) Δ-**1** in the presence of **htel(Na)** in 50 mM Na-phosphate, pH 7, in D_2O (λ_{ex}= 400 nm, 2 kHz,150 fs).

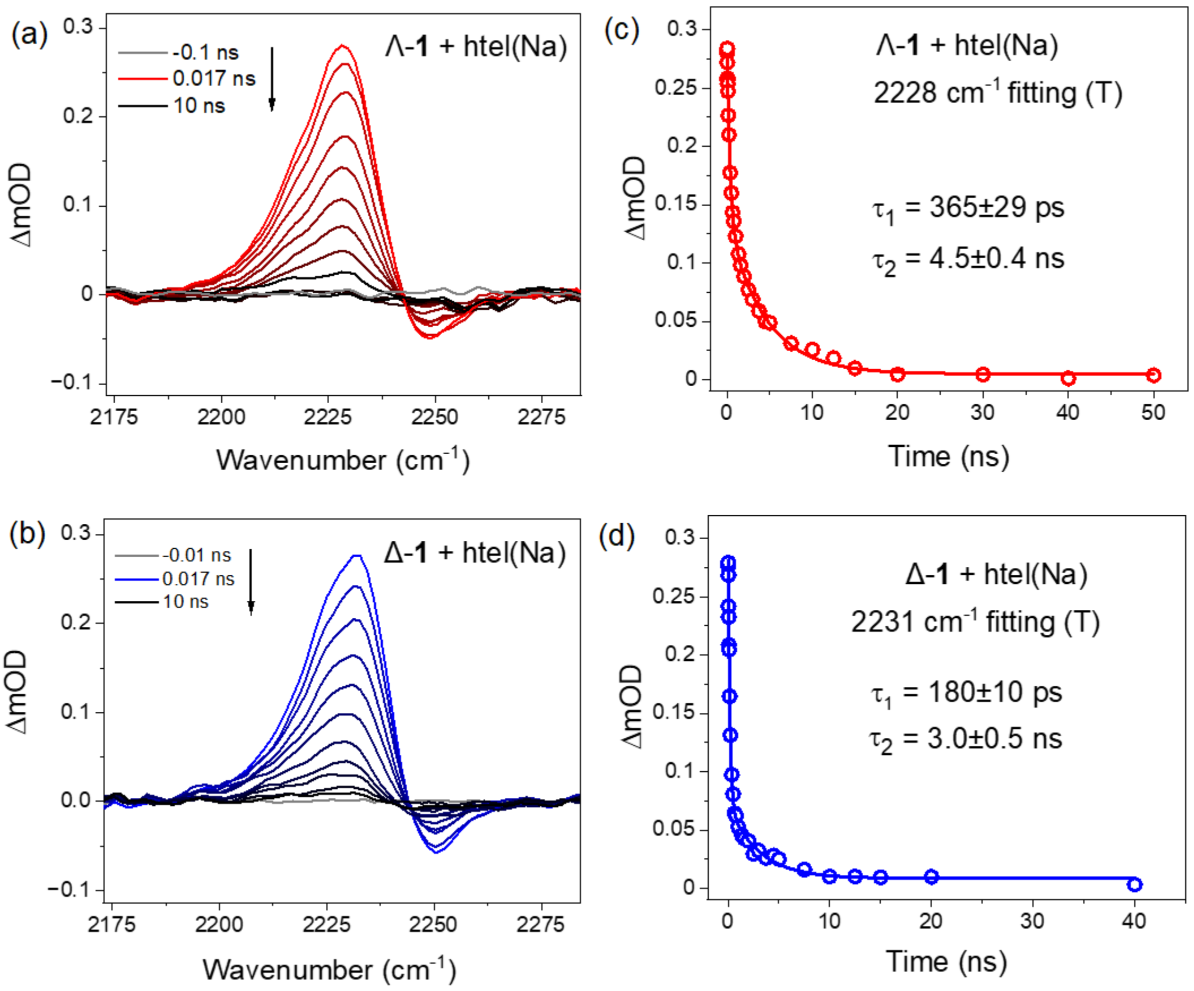

Figure S11: TRIR monitoring of the Nitrile band (a)/(c) Λ-**1** and (b)/(d) Δ-**1** when bound to **htel(Na)**.

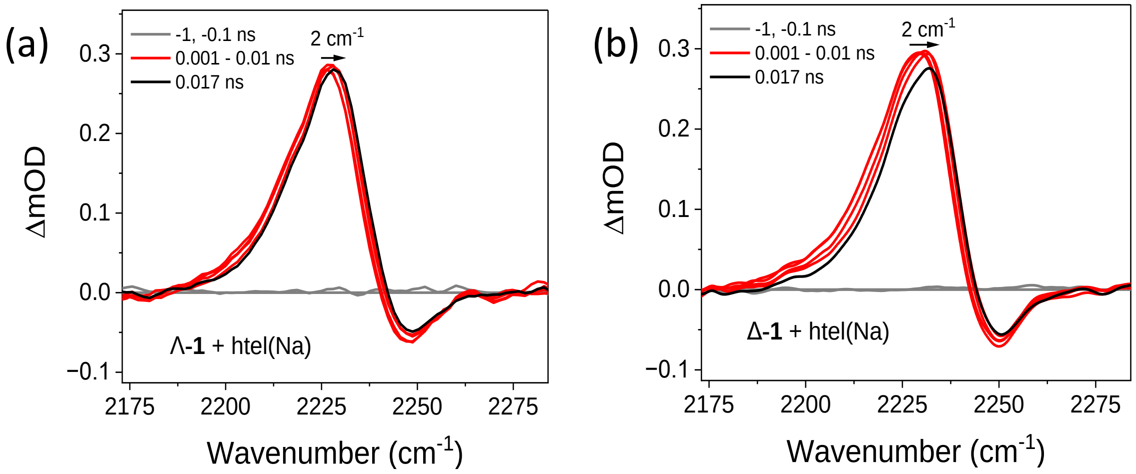

Figure S12: Vibrational cooling Nitrile band (a) Λ-**1** and (b) Δ-**1** when bound to **htel(Na)**.

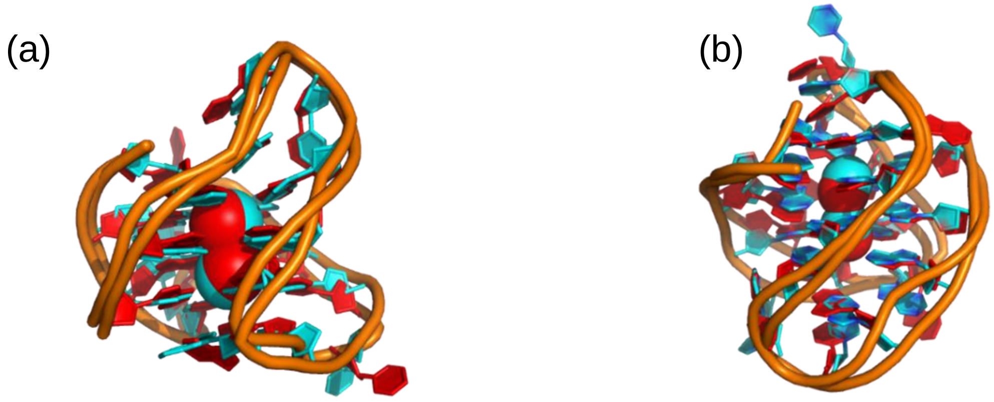

Figure S13: Human telomere sequence $d[AG_3(TTAG_3)_3]$ before (blue) and 100 ns after MD simulation (red) of (a) hybrid **htel(K)** PDB: 2HY9[5] and (b) anti-parallel **htel(Na)** PDB 143D[6].

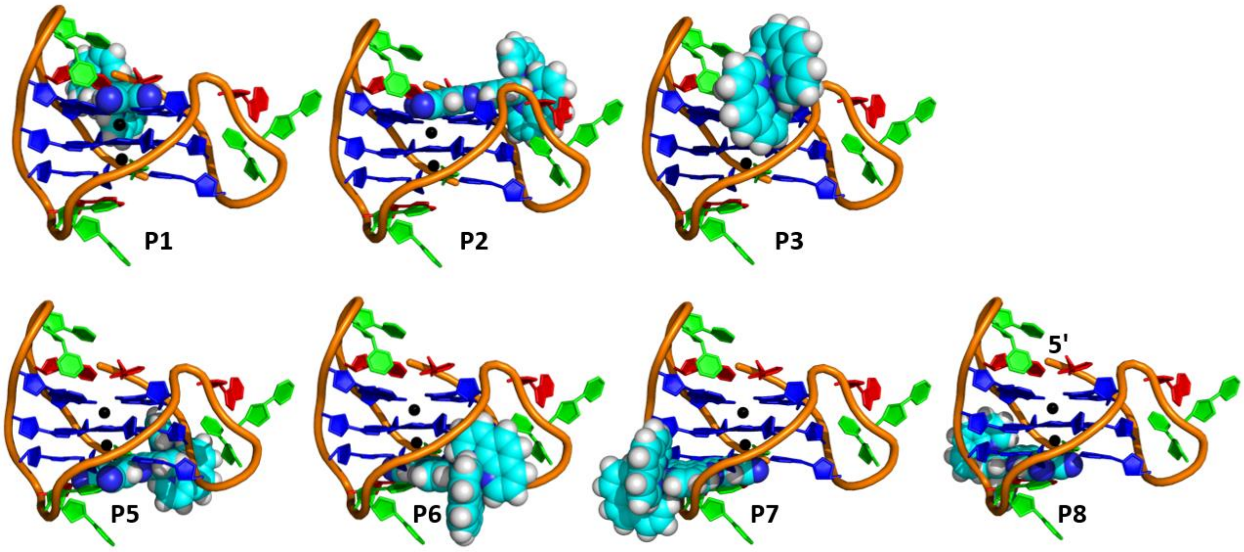

Figure S14: Explored human telomere sequence binding modes of Δ-**1** and hybrid **htel(K)** PDB 2HY9[5]

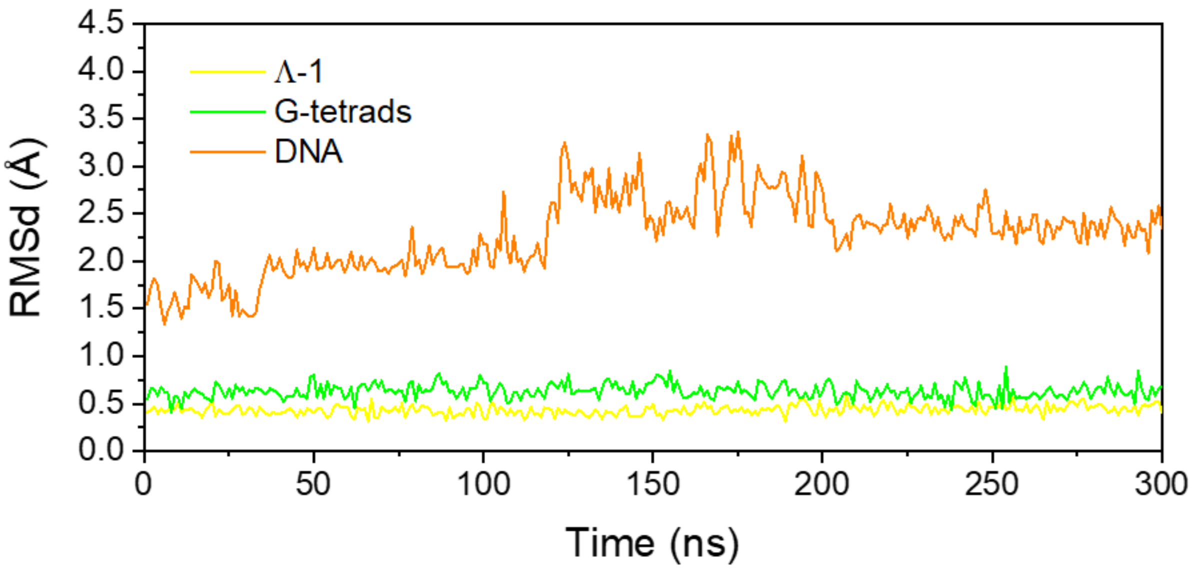

Figure S15: RMSD analysis of the binding of Λ-**1** to **htel(K)** in **P7** from the initial starting conformation of the MD simulation over the 350 ns duration of the simulation. The complex is shown in yellow, the G-tetrad (green) and the quadruplex (orange).

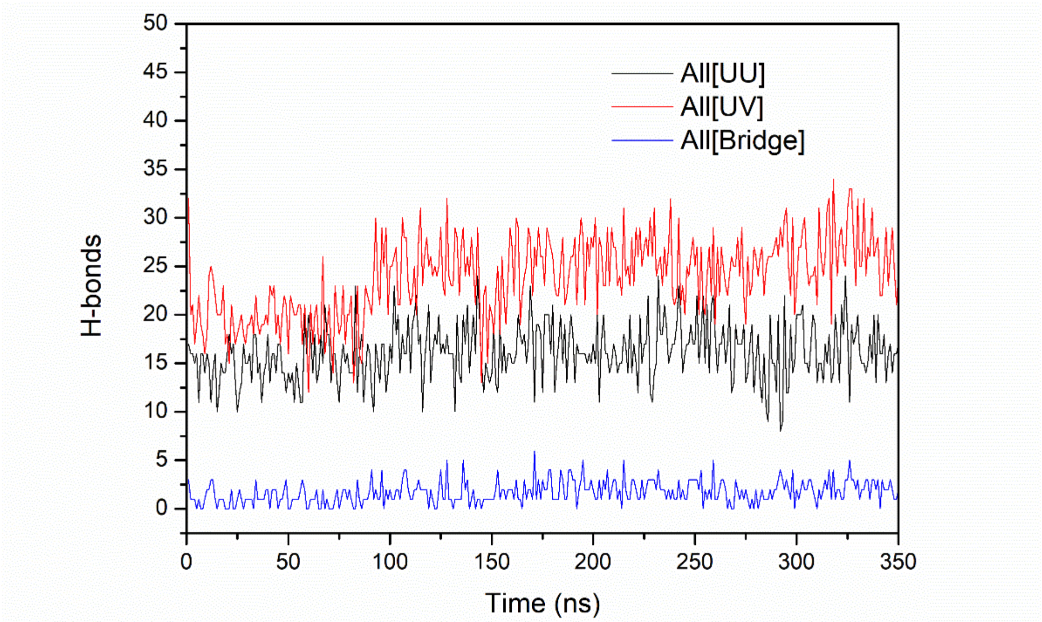

Figure S16: The variation in H-bonds over the 350 ns simulation period of Λ-**1** and hybrid **htel(K)** bound to **P7**. UU indicate solute-solute H-bonds (black), UV indicates solute-solvent H-bonds (red) and bridge indicates solute-solvent-solute bridges (blue).

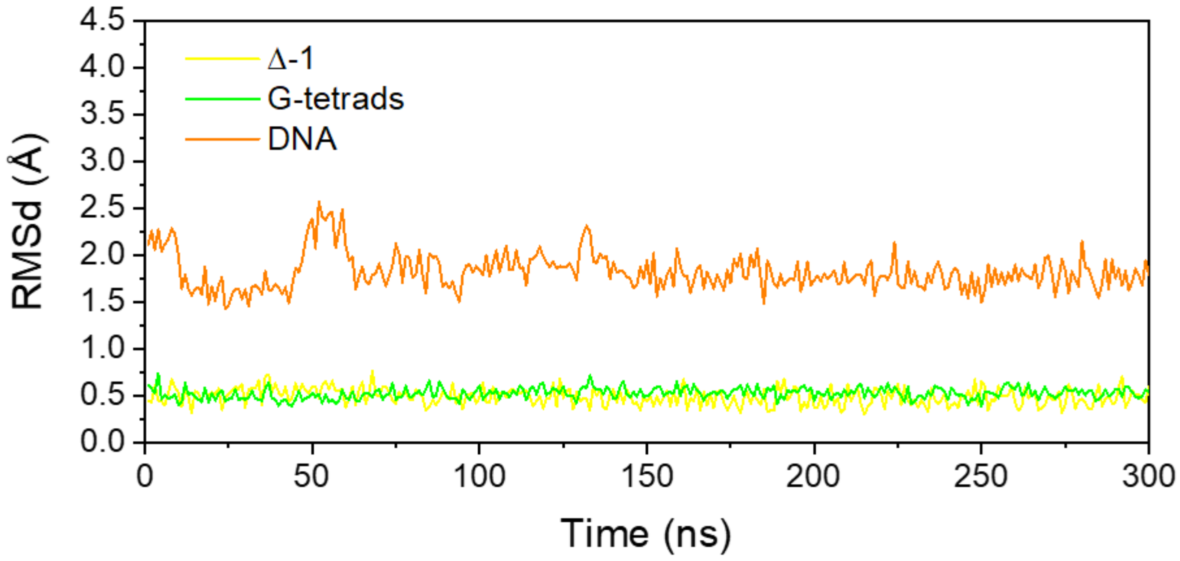

Figure S17: RMSD analysis of the binding of Δ-**1** to **htel(K)** in **P2** from the initial starting conformation of the MD simulation over the 350 ns duration of the simulation. The complex is shown in yellow, the G-tetrad (green) and the quadruplex (orange).

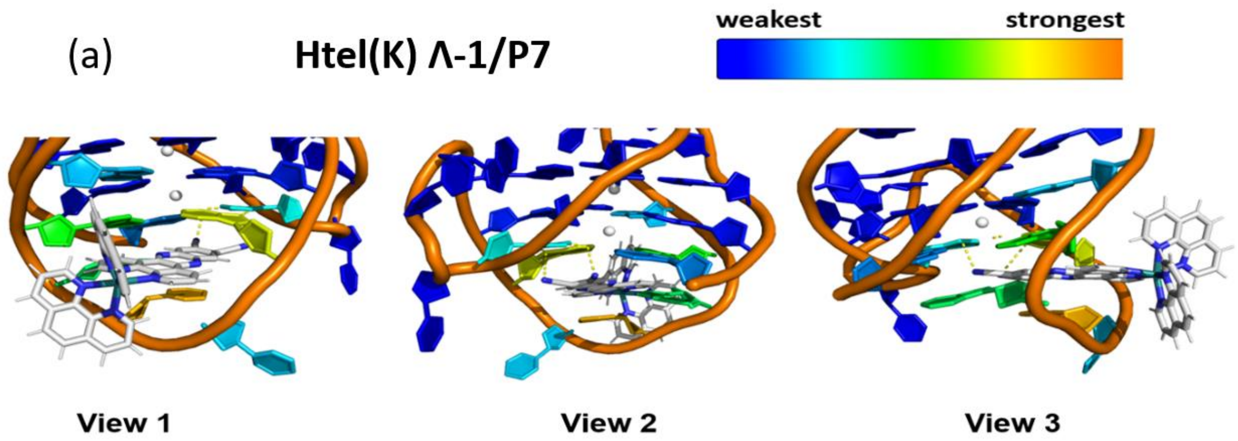

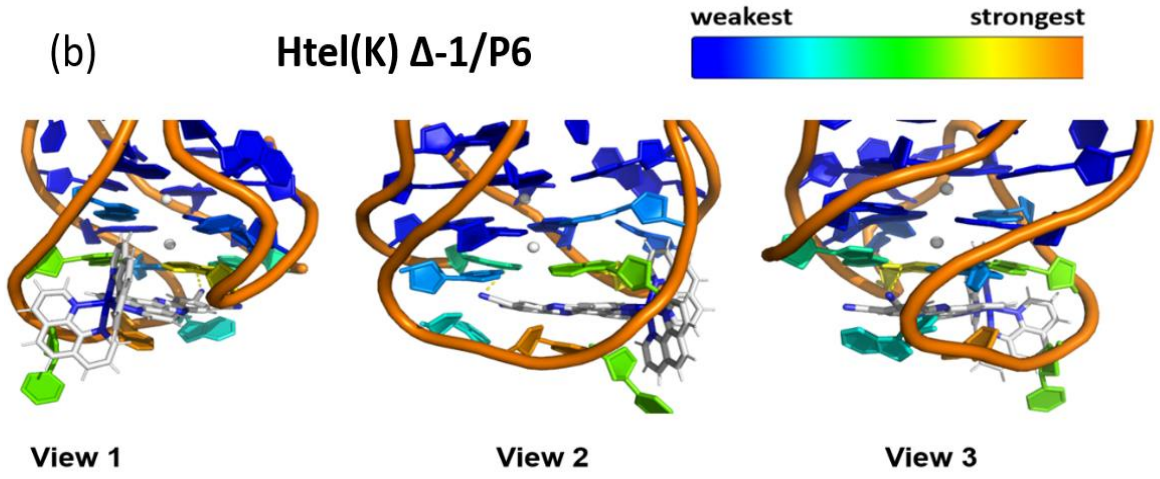

Figure S18: Additional views of enantiomer binding of (a) Λ-**1** (**P7**) and (b) Δ-**1** (**P6**) to hybrid **htel(K)** (PDB: 2HY9), highlighting nucleobase interactions from different views.

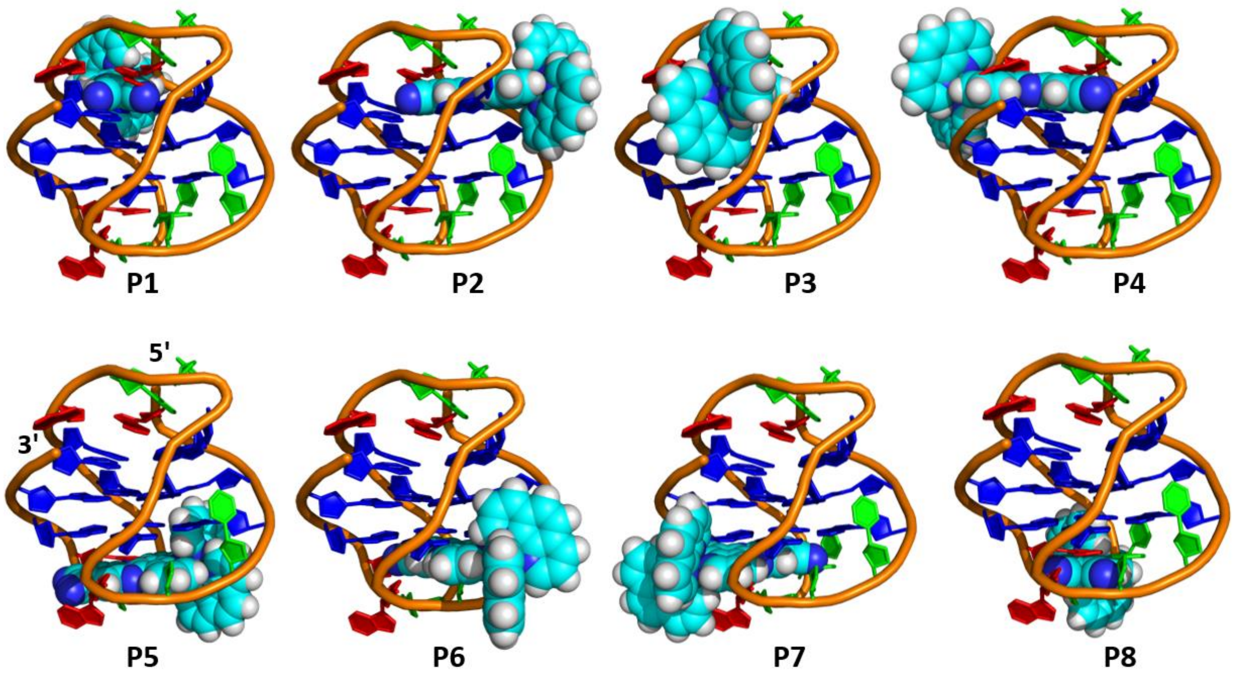

Figure S19: Feasible human telomere sequence binding modes of Δ-**1** and anti-parallel **htel(Na)** PDB 143D.

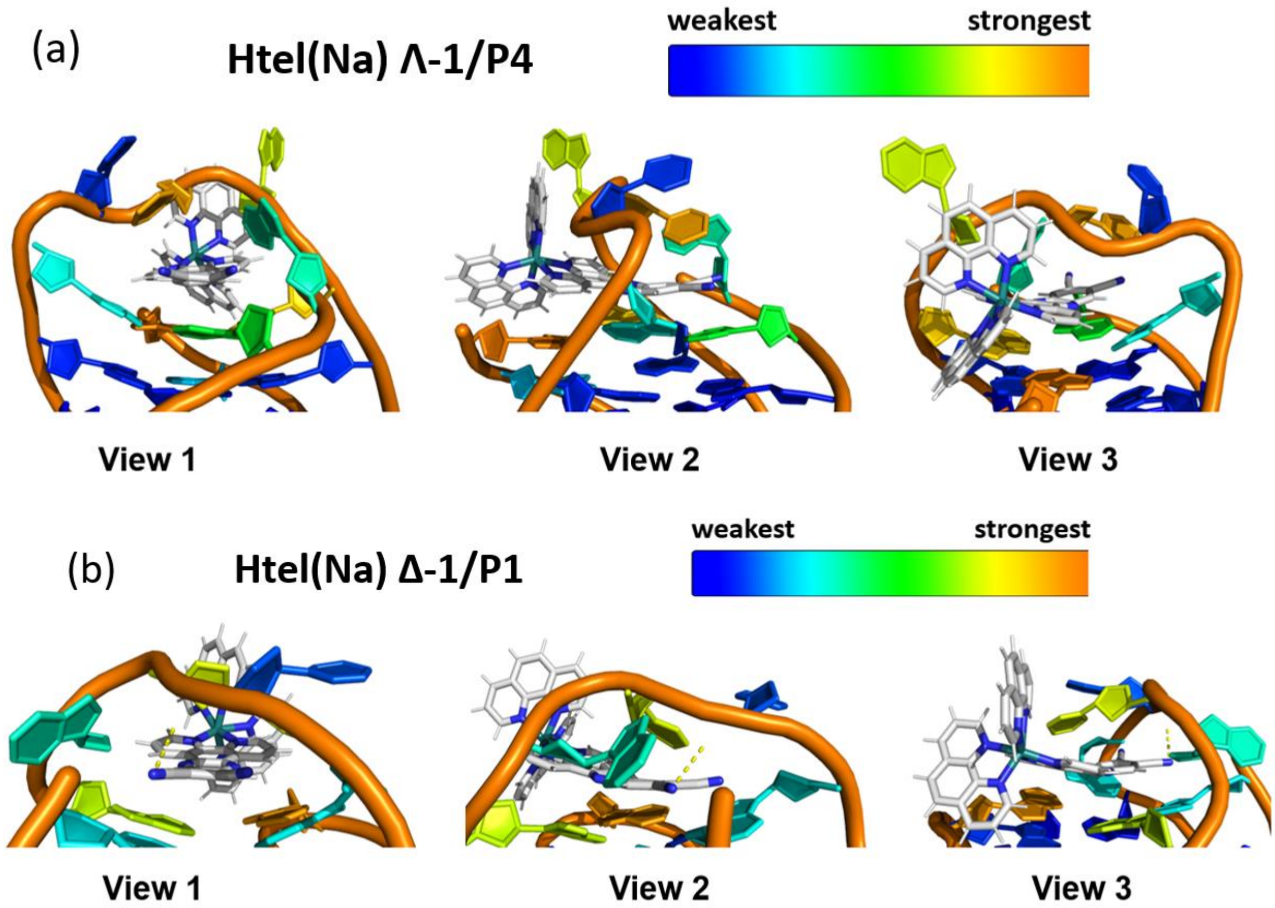

Figure S20: Additional views of enantiomer binding of (a) Λ-**1** (**P4**) and (b) Δ-**1** (**P1**) to anti-parallel **htel(Na)** (PDB: 143D), highlighting nucleobase from different views.

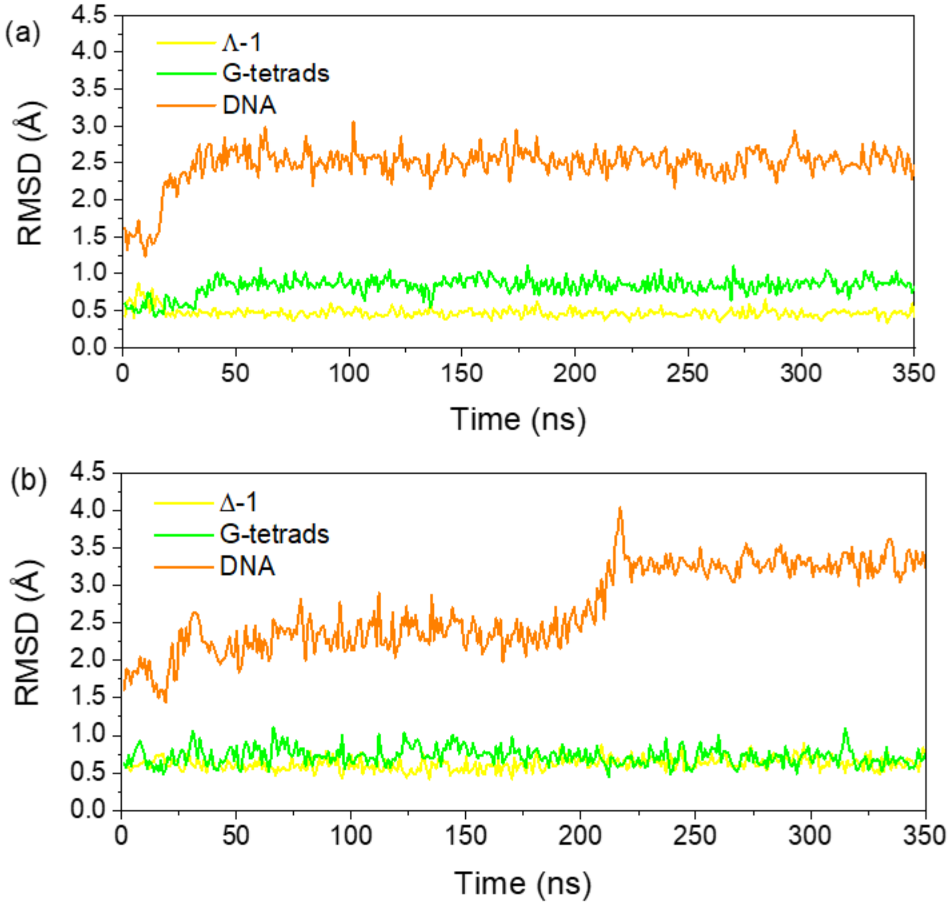

Figure S21: RMSD analysis of the binding of (a) Λ-**1** (**P4**) and (b) Δ-**1** (**P1**) to anti-parallel **htel(Na)** from the initial starting conformation of the MD simulation over the 350 ns duration of the simulation. The complex is shown in yellow, the G-tetrad (green) and the quadruplex (orange).

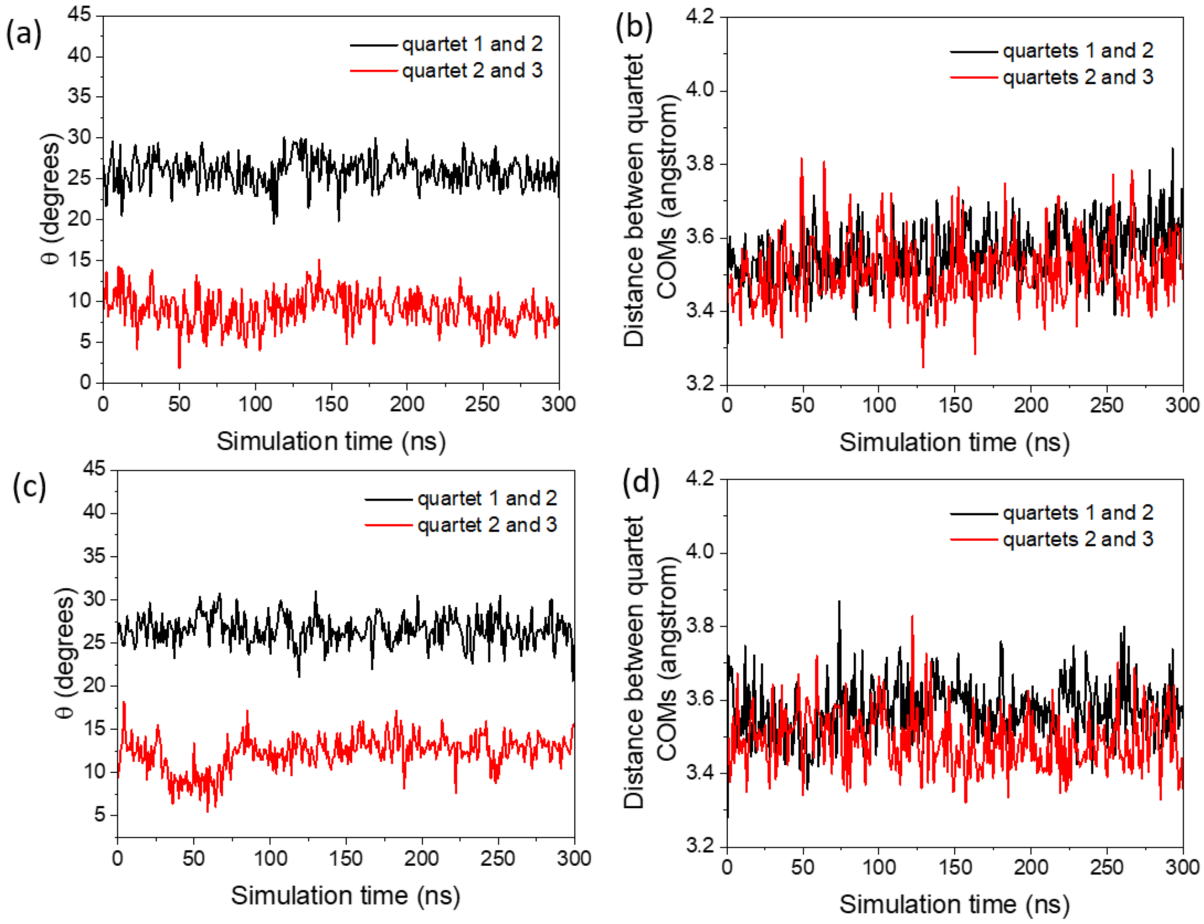

Figure S22: Computed fluctuations in the **htel(K)** G-tetrad structural parameters obtained using the method of Tsetkov et al.[7] to describe conformational rearrangements in G quadruplexes. (a) Twist angle variation and (b) distances between quartet centres of mass (COMs) for Λ-**1**/**P7** and (c) twist angle variation and (d) distances between quartet centres of mass (COMs) for Δ-**1**/**P2**.

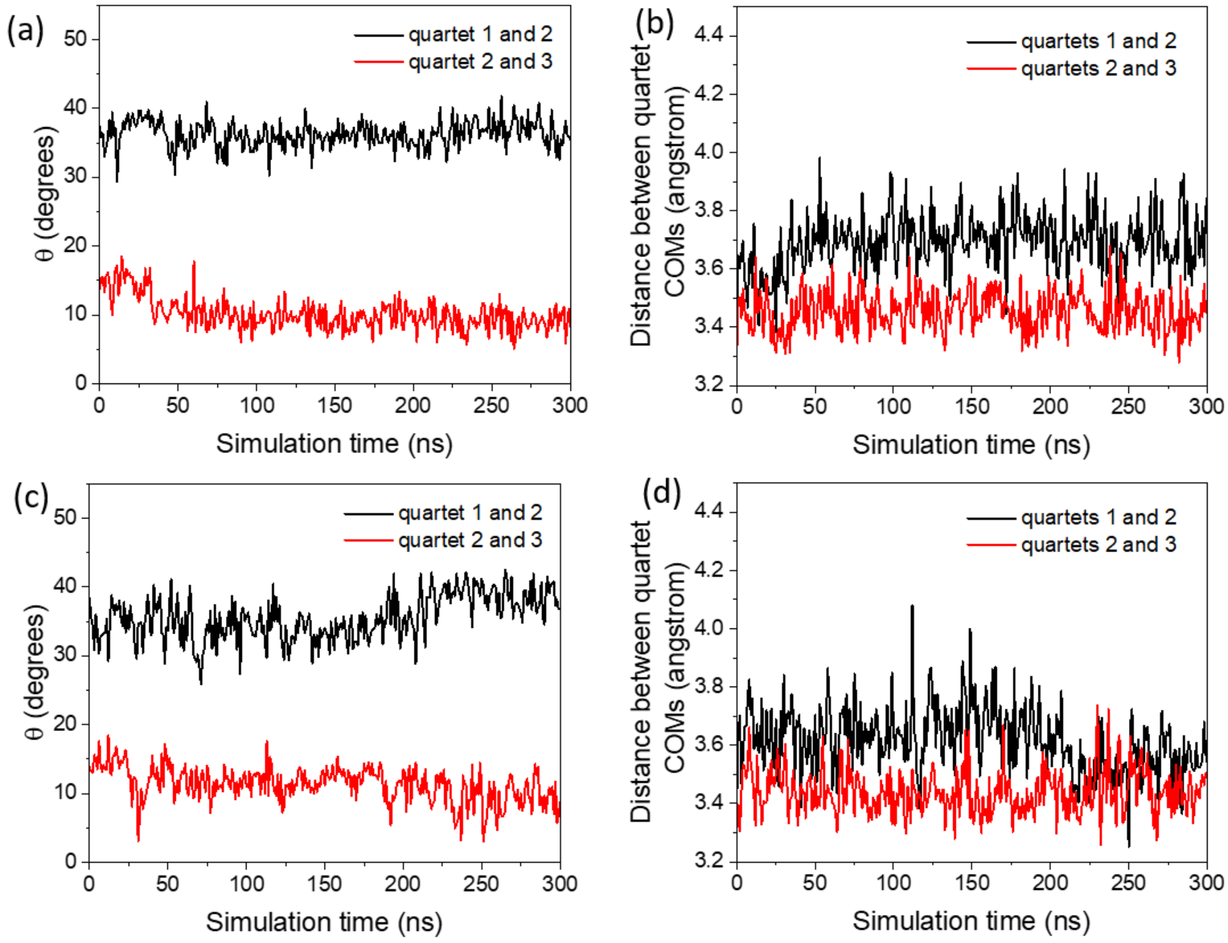

Figure S23: Computed fluctuations in the **htel(Na)** G-tetrad structural parameters obtained using the method of Tsetkov et al.[7] to describe conformational rearrangements in G quadruplexes. (a) Twist angle variation and (b) distances between quartet centres of mass (COMs) for Λ-**1**/**P4** and (c) twist angle variation and (d) distances between quartet centres of mass (COMs) for Δ-**1**/**P1**.

Tables

Table S1. Summarized photophysical data for **1**.

	λ_{abs} / nm(ε / mol^{-1}dm^{3}cm^{-1})
**1**	440 (21,000), 380 (23,500), 264 (118,000)

Table S2: Wavenumber bleach and transient peak values and calculated wavenumber difference between peaks.

Solvent	Wavenumber (cm^{-1}) Bleach (B)	Wavenumber (cm^{-1}) Transient (T)	Λν(C≡N) / (cm^{-1})
H_2O	2251	2233	18
25%DMSO	2249	2229	20
50%DMSO	2246	2226	20
75%DMSO	2243	2222	21
100%DMSO	2240	2219	21
Λ-1 + htel(K)	2249	2229	20
Δ-1 + htel(K)	2248	2228	20
Λ-1 + htel(Na)	2249	2228	21
Δ-1 + htel(Na)	2250	2231	19

Table S3: Λ-**1** binding positions investigated binding to hybrid G-quadruplex (PDB: 2HY9). The final 20 ns of the 100 ns simulation was used for MM-ISMSA analysis. All in (kcal mol^{-1}). VdW = Van der Waals, L deslov. = ligand desolvation R desolv. = Receptor desolvation

**Position**	**VdW**	**Electrostatics**	**L desolv.**	**R desolv.**	**Apolar**	**ΔG$_{binding}$**
**1**	-69.51	-4.52	0.24	11.14	-2.54	-65.19
**2**	-70.14	-5.60	0.37	14.19	-3.07	-70.29
**3**	-74.99	-4.02	0.21	11.80	-2.78	-69.78
**5**	-	-	-	-	-	-
**6**	-73.70	-6.16	0.65	14.06	-3.04	-68.18
**7**	-91.10	-5.42	0.27	11.74	-3.36	-87.87
**8**	-	-	-	-	--	-

Table S4: **Λ-1** binding in **P7** binding to hybrid G-quadruplex (PDB: 2HY9). Investigated by simulating for threeindependent 350 ns MD simulation, the final 20 ns was used for MM-ISMSA analysis. All in (kcal mol^{-1}). VdW = Van der Waals, L deslov. = ligand desolvation R desolv. = Receptor desolvation

**Simulation**	**VdW**	**Electrostatics**	**L desolv.**	**R desolv.**	**Apolar**	**$\Delta G_{binding}$**
1	-82.82	-5.90	0.32	11.57	-2.94	-79.76
2	-69.56	-5.51	0.34	10.45	-2.54	-66.83
3	-82.17	-6.02	0.31	10.86	-2.93	-79.96

Table S5: **Δ-1** binding positions investigated binding to hybrid **htel(K)** (PDB: 2HY9). The final 20 ns of the 100 ns simulation was used for MM-ISMSA analysis. All in (kcal mol^{-1}). VdW = Van der Waals, L deslov. = ligand desolvation R desolv. = Receptor desolvation

**Position**	**VdW**	**Electrostatics**	**L desolv.**	**R desolv.**	**Apolar**	**$\Delta G_{binding}$**
**1**	-58.71	-4.59	1.74	12.08	-2.44	-51.90
**2**	-64.06	-5.76	1.16	9.85	-2.97	-61.78
**3**	-31.69	-3.92	0.93	6.86	-1.57	-29.38
5	-60.85	-5.26	1.22	9.37	-2.30	-57.82
6	-83.24	-5.37	1.80	12.34	-3.35	-77.82
7	-61.99	-4.69	2.21	7.03	-2.38	-59.82
8	-61.25	-5.38	1.17	9.20	-2.30	-58.56

Table S6: **Δ-1** binding in **P6** binding to hybrid **htel(K)** (PDB: 2HY9). Investigated by simulating for three independent 350 ns MD simulation, the final 20 ns was used for MM-ISMSA analysis. All in (kcal mol^{-1}). VdW = Van der Waals, L deslov. = ligand desolvation R desolv. = Receptor desolvation

**Simulation**	**VdW**	**Electrostatics**	**L desolv.**	**R desolv.**	**Apolar**	**$\Delta G_{binding}$**
1	-80.44	-5.31	1.75	13.22	-3.17	-73.95
2	-81.90	-5.17	1.71	13.70	-3.22	-74.88
3	-79.81	-5.95	1.41	14.97	-3.16	-72.54

Table S7: **Λ-1** binding positions investigated binding to antiparallel **htel(Na)** (PDB: 143D). The final 20 ns of the 100 ns simulation was used for MM-ISMSA analysis. All in (kcal mol^{-1}). VdW = Van der Waals, L deslov. = ligand desolvation R desolv. = Receptor desolvation

Position	VdW	Electrostatics	L desolv.	R desolv.	Apolar	$\Delta G_{binding}$
1	-58.02	-11.27	0.35	8.29	-2.11	-62.76
2	-64.15	-11.89	0.4	8.89	-2.47	-69.31
3	-69.29	-11.48	0.3	10.08	-2.37	-72.74
4	-90.97	-13.91	0.39	13.9	-3.41	-94.00
**5**	-55.52	-11.5	0.36	10.13	-2.11	-55.64
6	-55.16	-12.88	0.45	10.46	-2.26	-59.39
7	-	-	-	-	-	-
8	-65.7	-12.01	0.32	8.75	-2.51	-71.16

Table S8: **Δ-1** binding positions investigated binding to antiparallel **htel(Na)** (PDB: 143D). The final 20 ns of the 100 ns simulation was used for MM-ISMSA analysis. All in (kcal mol^{-1}). VdW = Van der Waals, L deslov. = ligand desolvation R desolv. = Receptor desolvation

Position	VdW	Electrostatics	L desolv.	R desolv.	Apolar	$\Delta G_{binding}$
1	-75.39	-12.69	2.09	8.61	-2.87	-80.24
2	-75.46	-12.32	2.34	10.64	-3.20	-78.00
3	-47.17	-11.60	1.36	7.82	-1.99	-51.58
4	-70.04	-11.72	2.04	7.30	-2.36	-74.78
**5**	-64.03	-10.85	1.86	10.27	-2.43	-65.19
6	-67.98	-12.20	1.49	9.55	-2.38	-71.51
7	-	-	-	-	-	-
8	-65.62	-11.87	1.91	7.82	-2.61	-70.37

Table S9: **Λ-1** enantiomer in the singlet state binding in **P4** to **htel(Na)** (PDB: 143D). Investigated by simulating for an independent 350 ns MD simulation, the final 20 ns was used for MM-ISMSA analysis. All in (kcal mol^{-1}).

Simulation	vdW	Electrostatics	L desolv.	R desolv.	Apolar	$\Delta G_{binding}$ (Kcal/mol)
1	-92.34	-13.87	0.40	13.59	-3.44	-95.65
2	-69.65	-11.77	0.21	10.70	-2.43	-72.96
3	-62.92	-11.31	0.28	10.09	-2.22	-66.09

Table S10: Δ-**1** enantiomer in the singlet state binding in **P1** to **htel(Na)** (PDB: 143D). Investigated by simulating for three independent 350 ns MD simulation, the final 20 ns was used for MM-ISMSA analysis. All in (kcal mol^{-1}).

Simulation	vdW	Electrostatics	L desolv.	R desolv.	Apolar	$\Delta G_{binding}$ (Kcal/mol)
1	-70.19	-12.58	2.07	8.88	-2.65	-74.47
2	-47.93	-10.81	1.59	9.33	-2.24	-50.06
3	-52.20	-11.70	1.94	8.16	-2.07	-55.87

Table S11: Analysis of the integrity of Hydrogen bonds along the tetrad trajectories for **htel(K)** Λ-**1**/**P7**.

DONOR	ACCEPTOR	Occur.
tetrad 1		
DG6-Side-N1	DG24-Side-O6	44.70%
DG6-Side-N2	DG24-Side-N7	24.50%
DG12-Side-N1	DG6-Side-O6	33.77%
DG12-Side-N2	DG6-Side-N7	24.50%
DG20-Side-N1	DG12-Side-O6	45.70%
DG20-Side-N2	DG12-Side-N7	22.85%
DG24-Side-N1	DG20-Side-O6	23.18%
DG24-Side-N2	DG20-Side-N7	25.50%
tetrad 2		
DG7-Side-N1	DG13-Side-O6	0.33%
DG7-Side-N2	DG13-Side-N7	26.16%
DG13-Side-N1	DG19-Side-O6	5.30%
DG13-Side-N2	DG19-Side-N7	42.72%
DG19-Side-N1	DG25-Side-O6	0.66%
DG19-Side-N2	DG25-Side-N7	46.69%
DG25-Side-N1	DG7-Side-O6	1.32%
DG25-Side-N2	DG7-Side-N7	45.36%
tetrad 3		
DG8-Side-N1	DG14-Side-O6	36.09%
DG8-Side-N2	DG14-Side-N7	27.48%
DG14-Side-N1	DG18-Side-O6	23.84%
DG14-Side-N2	DG18-Side-N7	38.08%
DG18-Side-N1	DG326-Side-O6	28.15%
DG18-Side-N2	DG326-Side-N7	25.83%
DG26-Side-N1	DG8-Side-O6	28.81%
DG26-Side-N2	DG8-Side-N7	25.83%

Table S12: Analysis of the integrity of Hydrogen bonds along the tetrad trajectories for **htel(K)** Δ-**1**/**P2**.

DONOR	Acceptor	occur.
tetrad 1		
DG6-Side-N1	DG24-Side-O6	19.94%
DG6-Side-N2	DG24-Side-N7	34.76%
DG12-Side-N1	DG6-Side-O6	40.74%
DG12-Side-N2	DG6-Side-N7	30.77%
DG20-Side-N1	DG12-Side-O6	38.46%
DG20-Side-N2	DG12-Side-N7	25.36%
DG24-Side-N1	DG20-Side-O6	34.47%
DG24-Side-N2	DG20-Side-N7	20.80%
tetrad 2		
DG7-Side-N1	DG13-Side-O6	5.13%
DG7-Side-N2	DG13-Side-N7	38.46%
DG13-Side-N1	DG19-Side-O6	3.42%
DG13-Side-N2	DG19-Side-N7	52.71%
DG19-Side-N1	DG25-Side-O6	0.57%
DG19-Side-N2	DG25-Side-N7	37.61%
DG25-Side-N1	DG7-Side-O6	1.14%
DG25-Side-N2	DG7-Side-N7	31.62%
tetrad 3		
DG8-Side-N1	DG14-Side-O6	46.72%
DG8-Side-N2	DG14-Side-N7	22.79%
DG14-Side-N1	DG18-Side-O6	19.37%
DG14-Side-N2	DG18-Side-N7	34.19%
DG18-Side-N1	DG26-Side-O6	29.06%
DG18-Side-N2	DG26-Side-N7	30.20%
DG26-Side-N1	DG8-Side-O6	22.22%
DG26-Side-N2	DG8-Side-N7	39.60%

Table S13: Analysis of the integrity of Hydrogen bonds along the tetrad trajectories for **htel(Na) Δ-1/P4**.

DONOR	ACCEPTOR	Occur.
tetrad 1		
DG8-Side-N1	DG12-Side-O6	21.08%
DG8-Side-N2	DG12-Side-N7	36.47%
DG12-Side-N1	DG24-Side-O6	13.96%
DG12-Side-N2	DG24-Side-N7	46.15%
DG20-Side-N1	DG8-Side-O6	39.60%
DG20-Side-N2	DG8-Side-N7	33.62%
DG24-Side-N1	DG20-Side-O6	9.12%
DG24-Side-N2	DG20-Side-N7	50.43%
tetrad 2		
DG7-Side-N1	DG19-Side-N7	1.42%
DG7-Side-N2	DG19-Side-N7	24.22%
DG13-Side-N1	DG7-Side-O6	7.69%
DG13-Side-N2	DG7-Side-N7	54.13%
DG19-Side-N1	DG25-Side-O6	12.25%
DG19-Side-N2	DG25-Side-N7	32.48%
DG25-Side-N1	DG13-Side-O6	0.57%
DG25-Side-N2	DG13-Side-N7	11.68%
tetrad 3		
DG6-Side-N1	DG14-Side-O6	31.05%
DG6-Side-N2	DG14-Side-N7	12.25%
DG14-Side-N1	DG26-Side-O6	9.97%
DG14-Side-N2	DG26-Side-N7	57.55%
DG18-Side-N1	DG6-Side-O6	18.52%
DG18-Side-N2	DG6-Side-N7	46.44%
DG26-Side-N1	DG18-Side-O6	13.96%
DG26-Side-N2	DG18-Side-N7	25.93%

Table S14: Analysis of the integrity of Hydrogen bonds along the tetrad trajectories for **htel(Na) Δ-1/P1**.

DONOR	ACCEPTOR	Occur.
tetrad 1		
DG8-Side-N1	DG12-Side-O6	21.08%
DG8-Side-N2	DG12-Side-N7	36.47%
DG12-Side-N1	DG24-Side-O6	13.96%
DG12-Side-N2	DG24-Side-N7	46.15%
DG20-Side-N1	DG8-Side-O6	39.60%
DG20-Side-N2	DG8-Side-N7	33.62%
DG24-Side-N1	DG20-Side-O6	9.12%
DG24-Side-N2	DG20-Side-N7	50.43%
tetrad 2		
DG7-Side-N1	DG19-Side-N7	1.42%
DG7-Side-N2	DG19-Side-N7	24.22%
DG13-Side-N1	DG7-Side-O6	7.69%
DG13-Side-N2	DG7-Side-N7	54.13%
DG19-Side-N1	DG25-Side-O6	12.25%
DG19-Side-N2	DG25-Side-N7	32.48%
DG25-Side-N1	DG13-Side-O6	0.57%
DG25-Side-N2	DG13-Side-N7	11.68%
tetrad 3		
DG6-Side-N1	DG14-Side-O6	31.05%
DG6-Side-N2	DG14-Side-N7	12.25%
DG14-Side-N1	DG26-Side-O6	9.97%
DG14-Side-N2	DG26-Side-N7	57.55%
DG18-Side-N1	DG6-Side-O6	18.52%
DG18-Side-N2	DG6-Side-N7	46.44%
DG26-Side-N1	DG18-Side-O6	13.96%
DG26-Side-N2	DG18-Side-N7	25.93%

References

1. McQuaid, K.; Abell, H.; Gurung, S. P.; Allan, D. R.; Winter, G.; Sorensen, T.; Cardin, D. J.; Brazier, J. A.; Cardin, C. J.; Hall, J. P., Structural Studies Reveal Enantiospecific Recognition of a DNA G-Quadruplex by a Ruthenium Polypyridyl Complex. *Angew Chem Int Ed Engl* **2019**, *58*, 9881-9885.
2. Fairbanks, S. D.; Robertson, C. C.; Keene, F. R.; Thomas, J. A.; Williamson, M. P., Structural Investigation into the Threading Intercalation of a Chiral Dinuclear Ruthenium(II) Polypyridyl Complex through a B-DNA Oligonucleotide. *J. Am. Chem. Soc.* **2019**, *141*, 4644-4652.
3. C. Fletcher, N.; Richard Keene, F., New synthetic route to monocarbonyl polypyridyl complexes of ruthenium: their stereochemistry and reactivity. *Dalton Trans* **1998**, 2293-2302.
4. Carter, M. T.; Rodriguez, M.; Bard, A. J., Voltammetric studies of the interaction of metal chelates with DNA. 2. Tris-chelated complexes of cobalt(III) and iron(II) with 1,10-phenanthroline and 2,2'-bipyridine. *J. Am. Chem. Soc.* **1989**, *111*, 8901-8911.
5. Dai, J.; Punchihewa, C.; Ambrus, A.; Chen, D.; Jones, R. A.; Yang, D., Structure of the intramolecular human telomeric G-quadruplex in potassium solution: a novel adenine triple formation. *Nucleic Acids Res* **2007,** *35*, 2440-50.
6. Wang, Y.; Patel, D. J., Solution structure of the human telomeric repeat d[AG3(T2AG3)3] G-tetraplex. *Structure* **1993,** *1*, 263-282.
7. Tsvetkov, V.; Pozmogova, G.; Varizhuk, A., The systematic approach to describing conformational rearrangements in G-quadruplexes. *J. Biomol. Struct. Dyn.* **2016,** *34*, 705-715.
